# Unveiling the potency of ZnO and CuO nanocomposites in combating hepatocellular carcinoma by inducing cell death and suppressing migration

**DOI:** 10.1038/s41598-025-97395-4

**Published:** 2025-05-03

**Authors:** Rasha M. Allam, Nesma M.E. Abo El-Nasr, Marawan A. Elbaset, Dalia O. Saleh, Ahmed M. A. El-Seidy

**Affiliations:** 1 Pharmacology Department, Medical Research and Clinical Studies Institute, National Research Centre, P.O. 12622, Cairo, Egypt; 2 Inorganic Chemistry Department, Advanced Materials Technology & Mineral Resources Research Institute, National Research Centre, P.O. 12622, Dokki, Cairo Egypt

**Keywords:** Zinc oxide (ZnO), Copper oxide (CuO), Bimetallic nanoparticles (BNPs), Sorafenib, HuH-7 cells, Autophagy, Migration, Cell death (Apoptosis, Necrosis), G2/M arrest, Cancer, Drug discovery, Molecular biology

## Abstract

Human hepatocellular carcinoma (HCC) is recognized as one of the leading causes of death globally and is resistant to several anticancer drugs. As a result, it is critical to identify more effective druggable therapies. Metal oxide nanoparticles (MO-NPs), especially nanocomposites, have recently received much attention owing to their potential applications in cancer therapy. In this study, we synthesized zinc oxide (ZnO) and copper oxide (CuO) nanocomposites in different ratios (N1, N2, and N3). We evaluated their cytotoxicity against two HCC cell lines (HepG2 and HuH-7) and one normal liver cell (BNL), compared with Sorafenib as a standard therapy. Then, we investigated the potential underlying mechanisms of anticancer action employing flow cytometry, migration assay, and western blot. The results showed that the nanocomposite with an equal ratio of both ZnO and CuO-NPs (N1) exhibited the highest cytotoxic activity on the HuH7 cell line while exerting no detrimental impact on normal rat liver epithelial cells. Further investigation into the toxicity mechanisms of N1 revealed three modalities of induced cell death (apoptotic, necrotic, and autophagic) along with S- and G2/M cell cycle arrest, suggesting mitotic catastrophe. Furthermore, N1 displayed potent anti-migratory activity, surpassing sorafenib, upregulated the protein level of autophagy marker beclin-1, while downregulated the protein level of EMT-marker vimentin. Overall, our findings showed that combining ZnO-NPs and CuO-NPs is more intriguing in combating HCC, providing prospective guidance for evolving liver cancer therapy employing bimetallic NPs.

**Supplementary Information:**

The online version contains supplementary material available at 10.1038/s41598-025-97395-4.

## Introduction

Liver cancer is one of the most fatal malignancies worldwide, with approximately 866,136 new cases diagnosed annually and an estimated mortality rate of 758,725 deaths, according to the GLOBOCAN 2022 report (International Agency for Research on Cancer, 2022)^1^, highlighting a significant global health crisis^2,3^. It is classified into primary and secondary types^4^. Secondary liver cancer, often referred to as hepatoblastoma, is relatively rare. In contrast, hepatocellular carcinoma (HCC) accounts for nearly 90% of primary liver cancers. It poses a significant threat to patient health, ranking fourth globally in cancer incidence and mortality^5^.

Despite advancements in treatment that have improved overall survival rates, high recurrence and metastasis rates remain the primary causes of mortality^6,7^. Epithelial–mesenchymal transition (EMT) plays a crucial role in liver cancer recurrence and metastasis. Thus, targeting EMT is essential for enhancing treatment outcomes in HCC patients^8^.

Chemotherapy remains a primary therapeutic strategy for HCC, with Sorafenib established as the first-line treatment for advanced stages^9,10^. However, its efficacy is limited by non-specificity and the development of resistance. Additionally, most HCC patients are diagnosed at advanced stages, where standard chemotherapy is often ineffective, underscoring the urgent need for more effective and targeted therapeutic strategies^11,12^.

Nanoparticles (NPs) have gained significant attention in cancer therapy, both preclinically and clinically, due to their high efficacy, safety, and ability to overcome resistance—one of the major challenges of conventional treatments^3,13,14^.

Several metal oxide nanoparticles (NPs) have been developed for both the diagnosis and treatment of malignancies^15^. Among these, zinc oxide nanoparticles (ZnO-NPs) have demonstrated remarkable anticancer properties, attributed to their high stability, inherent electrostatic nature, selective toxicity, biocompatibility, and unique physicochemical characteristics^16–19^.

Also, copper oxide nanoparticles (CuO-NPs) have gained significant interest recently due to their promising anticancer properties^20–22^. However, addressing the toxicity concerns associated with CuO-NPs is necessary due to their excessive reactive oxygen species (ROS) production that can induce oxidative stress, potentially endangering normal cells and vital organs^15,23^.

Bimetallic nanoparticles (BNPs), on the other hand, exhibit synergistic effects, enhanced stability, increased functionality, and a greater number of reactive sites^24,25^. As a result, their antitumor activity has been extensively evaluated in various types of cancer^24,26^. However, challenges remain, particularly in developing more efficient methods for nanoparticle synthesis and characterization^14,27^. Moreover, combining ZnO-NPs and CuO-NPs has been deemed an innovative and potential therapeutic agent for cancer treatment^28^.

Therefore, this study investigated the therapeutic potential of ZnO/CuO nanoparticles (N1, N2, and N3) at different ratios to evaluate their anticancer efficacy against hepatocellular carcinoma (HCC) in vitro. The most potent nanoparticle formulation was selected based on its cytotoxic effects. It was further assessed for its impact on key hallmarks of cancer progression, including cell viability, cell cycle regulation, apoptosis, autophagy modulation, and migratory behavior in HuH-7 cells. Additionally, western blot analysis was employed to elucidate the underlying molecular mechanisms, focusing on key signaling pathways involved in apoptosis, autophagy, and EMT, which play critical roles in HCC progression and treatment resistance.

## Materials and methods

### Sample Preparation for biological assays

#### General procedure

ZnO-CuO (50:50) nanocomposite (N1), ZnO-CuO (85:15) nanocomposite (N2), and ZnO-CuO (15:85) nanocomposite (N3) were synthesized using sonication method^29–31^. KOH (Sigma-Aldrich, reagent grade, 90%, flakes) and PEG (polyethylene glycol (8000 LR), 1 g) (Fisher scientific, BioReagents), were dissolved in 250 mL DD (double distilled water. Basic copper carbonate (Sigma-Aldrich-reagent grade) was added to this mixture under sonication (2 min, 20 cycles, 2 min puse). Zinc acetate monohydrate (Glentham Life Science, 99.999%) (250 mL DD) was then added to the reaction mixture under sonication (2 min, 15 cycles, 2 min puse). The mixture was put under stirring (1000 rpm) at 85 °C. The resulting solution was completely dried before being crushed into granules. The resulting powders were then crushed after being calcined at 600 °C for 4 h in an air environment in a furnace. The resulting powder was washed several times with DD and dried (@ 100 °C) in an air environment in a furnace.

#### Starting materials

N-1 [(CuO)_0.50_ (ZnO)_0.50_]: 1.4 g Copper (II) hydroxide carbonate (12.66 mmol), 2.74 g Zinc acetate monohydrate (12.48 mmol), 0.35 g KOH (6.24 mmol). N-2 [(CuO)_0.16_(ZnO)_0.84_]: 0.45 g Copper (II) hydroxide carbonate (2.03 mmol), 4.66 g Zinc acetate monohydrate (21.23 mmol), 0.5 g KOH (8.91 mmol). N-3 [(ZnO)_0.15_ (CuO)_0.85_]: 5.08 g Copper (II) hydroxide carbonate (22.98 mmol), 1.82 g Zinc acetate monohydrate (8.29 mmol), 1.2 g KOH (21.39 mmol).

### Experimental techniques

The structural, crystallite size of the sample was investigated using X-ray diffraction. The XRD patterns were obtained from an X’pert PRO diffractometer with a Cu-radiation (λ = 1.542Å) at 45 K.V. and 35 mA over the range of 2θ = 2°–60°, and the average size of the crystallites was calculated by Debye–Scherrer equation. HR-TEM was carried out using the TEM model JEOL 2100 LB6 transmission electron microscope at the National Research Center, Cairo, Egypt. XPS was collected on K-ALPHA (Thermo Fisher Scientific, USA) with monochromatic X-ray Al K-alpha radiation − 10 to 1350 e.v spot size 400 μm at pressure 10 − 9 mbar with full spectrum pass energy 200 e.v and at narrow spectrum 50 e.v. ImageJ was used on TEM to obtain histogram data. Sonication condition: direct immersion, Ultrasonics vibracell, 20 kHz, 50% of 550 wt., temperature below 80 °C.

#### Cell culture

Human hepatocellular carcinoma cell lines **(HepG2)** and **(HuH-7)** and normal hepatocyte cell line **(BNL)** were obtained from Nawah Scientific Inc. (Mokattam, Cairo, Egypt). Cells were maintained in Dulbecco’s Modified Eagle Medium (DMEM, Gibco, USA) supplemented with 100 µg/mL of streptomycin (Lonza GmbH, Köln, Germany), 100 units/mL of penicillin (Lonza GmbH, Köln, Germany), and 10% fetal bovine serum (FBS; Gibco, NY, USA) at 5% CO2 and 37 °C.

### Biological studies

#### Cytotoxicity assay using SRB assay

To assess cell viability, the SRB assay was performed after treating HepG2, HuH-7, and BNL cells with the N1, N2, and N3. In 96-well plates, cells were seeded at a density of 1 × 10^3^ cells per well, and different concentrations of synthesized nanomaterials ranging from 10 to 1000 µg/mL were added to the cells after overnight incubation for 72 h. Sorafenib was used as a standard chemotherapy at lower concentration ranges from 0.01 to 30 µM. Later, the culture medium was aspirated, and cells were fixed for 1 h at 4 °C with 150 µL of 10% trichloroacetic acid (TCA) (Merck). This was followed by washing the cells five times with distilled water, 70 µL of sulforhodamine (SRB) solution (Sigma-Aldrich) (0.4% w/v) was added and incubated for 10 min at room temperature in a dark place. Cells were washed with 1% acetic acid (Chem-Lab) three times and allowed to air-dry. Then, 150 µL of Tris pH 10.5 (Chem-Lab) (10 mM) was added, and the absorbance was measured at 540 nm using a BMG LABTECH^®^-FLUOstar Omega microplate reader (Ortenberg, Germany). Half maximal inhibitory concentrations (IC50) values were calculated for each experiment using GraphPad Prism 6 software. IC50 values were reported as mean ± SD^32^.

### Cell cycle analysis

The cell cycle phases of HuH-7 cells (more sensitive cell line) were analyzed using Flow Cytometry after treatment with **N1** (the most potent nanomaterial), compared with sorafenib. In six-well plates, cells were seeded at a density of 1 × 10^4^ cells per well and incubated for 24 h. Then, the cells were treated with the predetermined IC50 values of N1 and Sorafenib for 48 h. After that, the cells were trypsinization, pelleted, washed twice, and resuspended with 1 mL of phosphate-buffered saline (PBS, Lonza GmbH, Köln, Germany). Then, the cells were fixed with 60% ethanol at 4°C for a minimum of 2 h. After washing with PBS, the pellets were treated with RNase (Sigma-Aldrich) and stained with Propidium Iodide (PI, Sigma-Aldrich) at 37 °C in the dark for 30 min. The cellular DNA content was determined using the ACEA NovocyteTM flow cytometer (ACEA Biosciences Inc., San Diego, CA, USA). The findings were evaluated using the ACEA NovoExpressTM software (ACEA Biosciences Inc., San Diego, CA, USA)^33^.

### Annexin V/PI apoptotic assay

The impact of N1 and sorafenib nanomaterial on the apoptotic/necrotic cell death of HuH-7 cells was investigated using the Annexin V-FITC Apoptosis Staining/Detection kit (ab14085; Abcam, Cambridge, MA). In brief, cells were planted in six-well plates at a density of 1 × 10^4^ cells per well for 24 h and exposed for treatment for another 48 h with the previously determined IC50 values. After that, the cells were trypsinized and washed twice with PBS. The cells were then resuspended in 500 µL of 1× Binding Buffer and stained with Annexin V-FITC and PI for 30 min at room temperature in the dark^34^.

### Autophagy assessment

For autophagic assessment in response to N1 and sorafenib treatment for 48 h, HuH-7 cells were trypsinized and washed twice with ice-cold PBS. Then 0.5 mL of the staining solution (1 µg/mL of acridine orange (Sigma-Aldrich, ≥ 98% HPLC), in PBS) was added and incubated in the dark for 30 min at room temperature. Cells were adjusted at 12,000 events when applied to flow cytometric analysis via ACEA Novocyte™ flow cytometer, and fluorescent signals were analyzed via FL1 signal detector (488 nm excitation/530 nm emission). The net fluorescent intensities (NFI) were quantified^35^.

### Scratch/wound healing assay

HuH-7 cells were seeded in 6-well culture plates at a seeding density of 3 × 10^5^/well to achieve the confluent monolayer. Then, the cell monolayer was gently scratched with a sterile 200-µL pipette tip to make one straight cell-free line. After PBS wash, cells were treated with sub-cytotoxic concentrations of N1 and sorafenib. Scratch healing was recorded at 0, 24, 48, 72, 96 and 120 h. The scratch images were captured at a magnification of ×100 using an inverted microscope (Olympus, Japan). The horizontal distance of the wound gap was measured using Image J (version 1.53 C, NIH, US). Experiments were carried out in triplicate. The percentage of wound closure was calculated according to the following equation: % Wound closure = 100 − [(Wt/W0) × 100], where *Wt* is the wound width at time t, and *W0* is its initial width^36^.

### Western blot

Cultured cells were harvested and lysed in a readymade RIPA buffer (Beyotime Biotechnology) containing a protease inhibitor cocktail (1 μg/mL aprotinin and leupeptin) to extract proteins from the cells. Proteins in the cell lysate (25 μg) were separated through SDS-PAGE gels and transferred to nitrocellulose membranes (Amersham Pharmacia Biotech, Buckinghamshire, UK). Membranes were blocked with 5% skimmed milk for 3 h; then, the membrane was incubated with the primary antibodies (all from FAGUS Antibody Services, UK) against Beclin-1 (1:1000), Vimentin (1:1000), E-Cadherin (1:1000) and GAPDH (1:1000), except for LC3-II (1:1000) (Cell signaling) at 4 °C overnight. The membrane was washed with TBS-T buffer and then incubated with the corresponding secondary antibodies at 37 °C for 2 h. Bands were visualized with the enhanced chemiluminescent (ECL/Thermo-Scientific) substrate according to the manufacturer’s recommendation. Chemiluminescence was detected using the Bio-Rad System (Bio-Rad gel electrophoresis system, Mini-PROTEAN^®^ Tetra Cell, and Mini Trans-Blot) and analyzed with the Bio-Rad ChemiDock Gel documentation imaging system^37^.

### Intracellular ROS measurement

The ROS level was assessed in HuH-7 cells after exposure to ZnO/CuO nanocomposite (N1). A monolayer of the cells was seeded in 6-well plates (1 × 10^4^ cells/well) and allowed to adhere for 24 h in a CO2 incubator at 37 °C. After that, cells were treated with N1 at different concentrations (5, 10, and 20 µg/mL) based on SRB cytotoxicity assay results for 24 h. After incubation, DCF reagent (2′,7′-Dichlorofluorescein) (Sigma-Aldrich, Bioreagent) was added to the wells. Triton-x100 (800 µL) was added to each well after incubating the plate for 45 min, washing it with PBS, and then kept in the refrigerator for half an hour. The cells were collected and centrifuged for 15 min. Finally, the fluorescence of the samples was measured at an excitation/emission wavelength of 488/510 nm using a microplate reader^38^.

## Results

### Characterization of nanocomposites

**Fig. 1:**
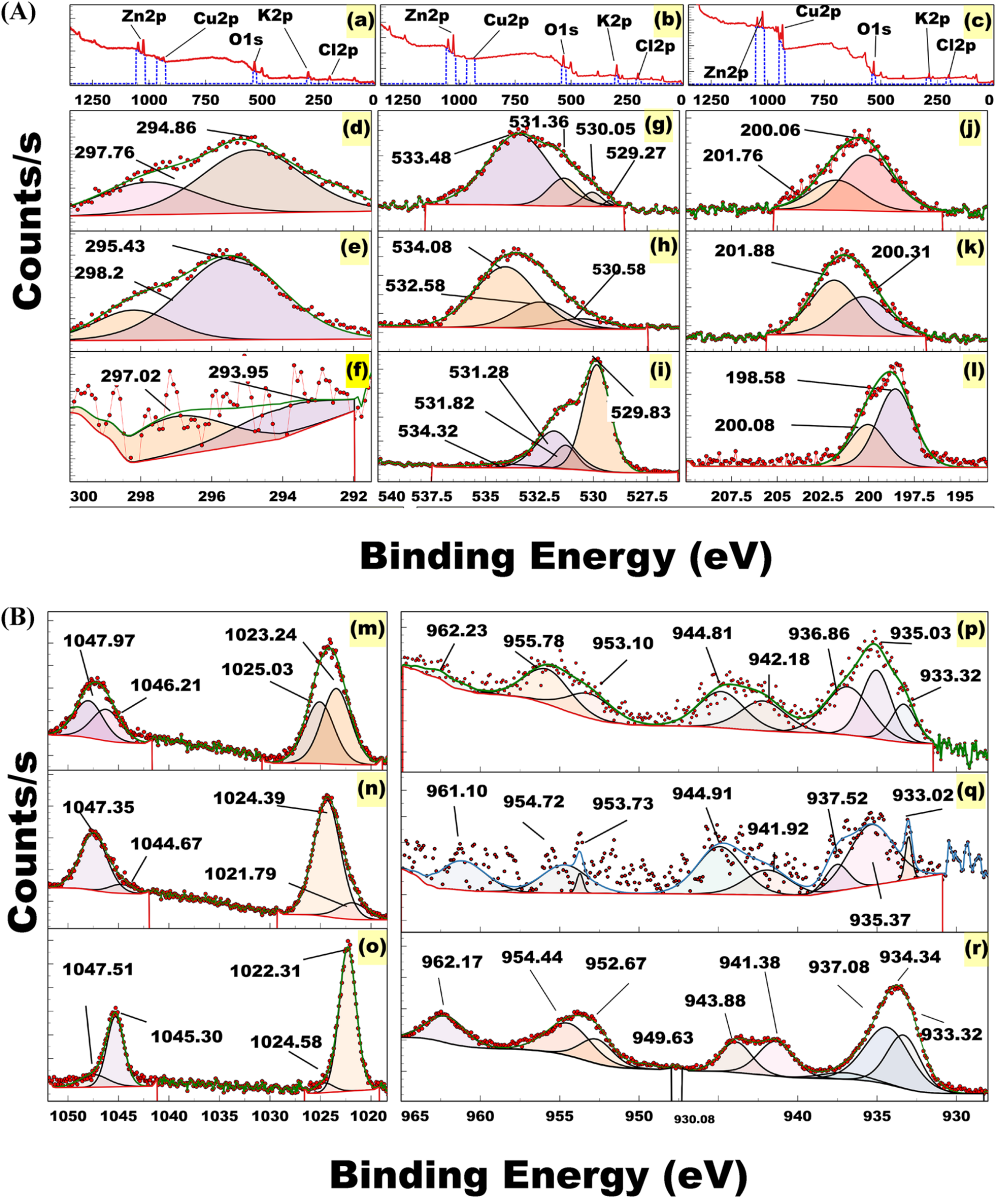
(**A**) XPS survey spectra of (a) ZnO-CuO (50:50) nanocomposite (N1), (b) ZnO-CuO (85:15) nanocomposite (N2), (c) ZnO-CuO (15:85) nanocomposite (N3); HR XPS K-2p spectra of (d) N1, (e) N2, (f) N3; HR XPS O-1s spectra of (g) N1, (h) N2, (i) N3; HR XPS Cl-2p spectra of (j) N1, (k) N2, (l) N3. (**B**) HR XPS Zn-2p spectra of (m) ZnO-CuO (50:50) nanocomposite (N1), (n) ZnO-CuO (85:15) nanocomposite (N2), (o) ZnO-CuO (15:85) nanocomposite (N3); and HR XPS Cu-2p spectra of (p) N1, (q) N2, (r) N3.

Figure 1a–c shows the survey spectrum of N1, N2, and N3, respectively. These spectra show peaks in 199.64–202.09, 296.19–296.74, 531.11–534.33, 934.5–937.31, and 1022.93–1024.85 eV regions, which were assigned to chlorine (Cl-2p), potassium (K-2p), oxygen (O-1s), copper (Cu-2p), and zinc (Zn-2p), respectively. The peaks of chlorine and potassium indicate the presence of KCl as a residue during the synthesis process. The presence of KCl helps to control particle size and increase the solubility of other oxides^31^. The fitted curves (Fig. 1m–o) were in good agreement with the experimental curves. Figure 1d–f and j–k show the HR K-2p and Cl-2p XPS spectra, respectively. These spectra show the characteristic peaks 2p_3/2_ and 2p_1/2_ for K^+^ (293.95–295.43, and 297.02–298.20 eV) and Cl^−^ (198.58–200.31 and 200.08–201.88 eV) supporting the presence of KCl^39^. HR O-1s XPS spectra (Fig. 1g–i) show peaks in 529.83–530.58 eV and 531.36–531.82 eV regions, which may be attributed to M-O and surface –OH group, respectively^40,41^. Figure 1g showed a peak at 533.48 eV, which may be attributed to the OH group^42^. Figure 1h showed a peak at 532.58 eV, while Fig. 1i showed a peak at 531.28 eV, which may be attributed to superoxide and chemisorbed oxygen species^43^. Figure 1h, i showed peaks in 534.08–534.32 eV, which may be attributed to the atmospheric water^39^. HR Zn-2p XPS spectra (Fig. 1d–f) showed ZnO.OH (Zn-2p_1/2_: 1047.47–1047.94 eV and Zn-2p_3/2_: 1024.34–1025.09 eV region)and ZnO (Zn-2p_1/2_: 1044.73–1046.21 eV and Zn-2p_3/2_: 1021.85–1023.44 eV region) characteristic peaks with a spin–orbits splitting value of ≈ 23 eV. The ratio of ZnO to the total quantity is calculated using Eq. (1). ZnO % were 52.04, 13.00, and 86.91 in N1, N2, and N3, respectively. The ZnO % is maximized in the nanocomposite with the lowest zinc content, which agrees with our previously reported results^29^.

1 $$\begin{aligned} \%\left[ZnO\right] &= \left(sum\: ZnO\:peaks\:area\:\right(2p3/2 + 2p1/2 \left) \times 100\right) \\&\quad /\left(sum\: ZnO\:peaks\:area\:\right(2p3/2 + 2p1/2 )\\&\quad + sum ZnO.OH\:peaks\:area\:(2p3/2 + 2p1/2\left)\right) \end{aligned}$$

HR Cu-2p XP spectra (Fig. 1q, r) show characteristic peaks (Cu-2p_3/2_ and Cu-2p_1/2_) of both Cu^2+^ (935.37–934.34 and 954.44–955.78 eV) and Cu^+^ (933.02–933.32 and 952.67–953.73 eV)^44,45^. The relative concentrations of CuO were calculated using Eq. (2). % CuO were 63.94%, 90.75%, and 61.18% for N1, N2, and N3, respectively. These spectra show characteristic peaks for the Cu^2+^ oxidation state in the 936.86–937.52 eV region, which corresponds to the Cu 3d^9^ shell in the 961.10–962.23 eV region, and known peaks in the 941.92–949.63 eV region^44,46^.

2 $$\begin{aligned} \%[Cu2+] &= (sum\:Cu2+\:peaks\:area\:(2p3/2 + 2p1/2\left) \times 100\right) / (sum\:Cu2\\ &\quad +\:peaks\:area\:(2p3/2 + 2p1/2) + sum\:Cu+\:peaks\:area\:(2p3/2 + 2p1/2 \left)\right) \end{aligned}$$

#### XRD

**Fig. 2:**
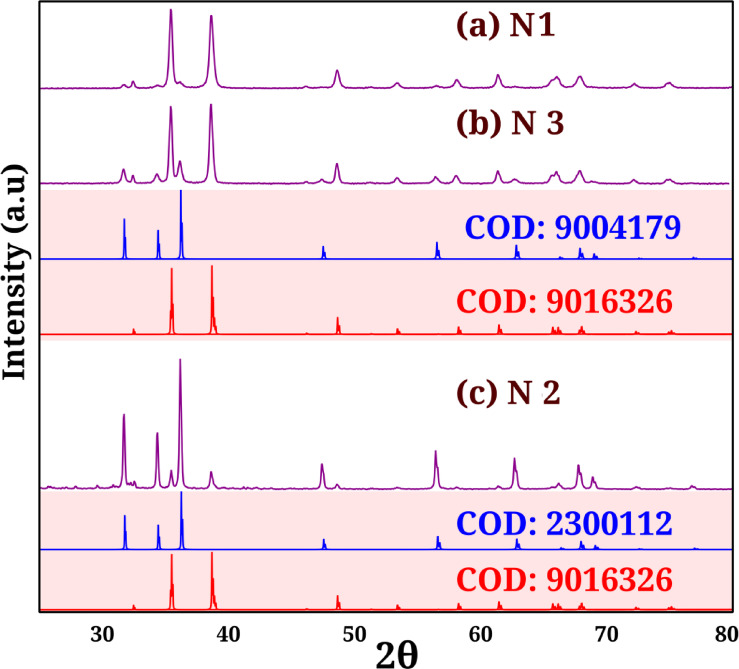
XRD spectra of (a) N-1 (b) N-3, (h) N-2 Nanocomposites and that obtained from COD literary.

The XRD spectra of N-1, N-2, and N-3 nanocomposites are shown in Fig. 2a–c, respectively, along with the spectra of CODs 9,016,326, 9,004,179, and 2,300,112. The most important peaks and their corresponding (h k l) values are given in Table 1. The calculated crystallite sizes and atomic parameters of Magnetite (B-1), Tenorite (B-1 and B-2), Magnetite (B-2), and Cuprospinel (B-3) are listed in Table 2.

The good crystallinity of N-1, N-2, and N-3 nanocomposites is supported by the sharp peak in the XRD spectra shown in Fig. 2a–c. The COD (Crystallography Open Database) was used as the starting point. All spectra show the presence of two phases, Tenorite (CuO) and Zincite (ZnO). The Zincite phase in N-1, N-2, and N-3 was matched by XRD reference codes COD: 9,004,179, 2,300,112, and 9,004,179, respectively, while that of the Tenorite phase was matched by XRD reference codes COD: 9,016,326. The XRD patterns of all spectra showed diffraction peaks for the cubic crystal system of Zincite with space group P 63 m c (186) and also the diffraction peaks for the monoclinic crystal system of Tenorite with space group C 1 c 1 (9), see Table 1. The crystallite sizes were calculated using Scherrer’s formula using FWHM (full-width half maximum), see Table 2.

Peaks at 35.36° (0.35, (3 1 1)), 38.58° (0.35, (1 1 1)), 38.59° (0.40, (1 1 1)), 35.42° (0.35, (3 1 1)), and 35.41° (0.35, (3 1 1)) were used to calculate the crystallite sizes, atomic and structural parameters of Magnetite (B-1), Tenorite (B-1 and B-2), Magnetite (B-2), and Cuprospinel (B-3), see Table 2.

**Table 1:** The most important XRD peaks of all phases and its corresponding h K L.

Tenorite (CuO)				Zincite (ZnO)			
	N1	N2	N3		N1	N2	N3
	COD: 9016326				COD: 9004179	COD: 2300112	COD: 9004179
hkl	Angle [2θ°]			hkl	Angle [2θ°]		
1 1 0	32.4314	32.4238	32.4295	1 0 0	31.6803	31.6788	31.6882
0 0 2	35.3256	35.327	35.3352	0 0 2	34.3278	34.3289	34.3203
1 1–1	35.4619	35.4515	35.4552	1 0 1	36.1527	36.1516	36.1579
1 1 1	38.6013	38.5986	38.6086	1 0–2	47.4046	47.4044	47.4045
2 0 0	38.7892	38.7892	38.8168	1 1 0	56.4287	56.4258	56.4436
1 1–2	46.1393	46.1287	46.1337	1 0–3	62.6711	62.6718	62.6651
2 0–2	48.599	48.5883	48.6068	2 0 0	66.1736	66.1701	66.1917
1 1 2	51.1768	51.1783	51.1935	1 1 2	67.7406	67.7387	67.7495
0 2 0	53.3675	53.3473	53.3408	1 1–2	68.8738	68.8706	68.8905
0 2 − 1	56.5884	56.5692	56.5643	0 0 4	72.344	72.3467	72.3264
2 0 2	58.0387	58.05	58.0856	2 0 2	76.7152	76.7125	76.7279
1 1–3	61.3633	61.3532	61.3618				
0 2–2	65.6343	65.6174	65.6168				
3 1–1	66.0206	66.0091	66.0449				
3 1 0	66.2375	66.233	66.2742				
1 1 3	67.6495	67.6543	67.6757				
2 2 0	67.9055	67.8879	67.901				
2 2 − 1	68.7093	68.6873	68.6972				
3 1–2	71.4695	71.4521	71.4839				
3 1 1	72.096	72.0988	72.146				
2 2 1	72.7158	72.7036	72.7218				
0 0 4	74.7211	74.7246	74.7441				
2 2–2	75.0483	75.0232	75.0322				
0 2–3	79.4899	79.4753	79.4808				

**Table 2:** The crystallite sizes and structural properties of zincite and Tenorite phases in nanocomposites (N1, N2, N3).

	Zincite			Tenorite		
	N-1	N-2	N-3	N-1	N-2	N-3
a Å (*)	3.26(3.25)	3.26(3.25)	3.26(3.25)	4.70 (4.69)	4.70 (4.69)	4.70 (4.69)
b Å (*)				3.43 (3.43)	3.43 (3.43)	3.43 (3.43)
c Å (*)	5.22(5.21)	5.22(5.21)	5.22(5.21)	5.15 (5.14)	5.15 (5.14)	5.15 (5.14)
2θ	36.15	36.15	36.17	38.6	35.45	35.46
FWHM	0.45	0.35	0.25	0.4	0.25	0.35
Crystallite size (nm)	18.61	23.88	33.43	21.04	33.36	23.83
Crystallite size (nm)	18.61	23.88	33.43	21.04	33.36	23.83
Microstrain	6.00	4.68	3.34	4.98	3.41	4.78
Specific surface area (Sm^2^g^−1^)	56.93	44.27	31.7	43.98	27.74	38.84
Axial ratio	1.60	1.60	1.60			
d_00x_ (Å)	2.82	3.26	2.82			
d_x00_ (Å)	2.61	2.61	2.61			
d_0 × 0_ (Å)	2.48	2.48	2.48			
Oxygen position parameter (× 10^2−^ )	37.99	37.99	37.98			
Zinc oxygen bond length (× 10^2−^)	19.57	19.57	19.57			

### Biological results

In this study, we investigated the in vitro effects of synthesized nanocomposites N1, N2, and N3 on human HCC cells using two cell lines (HepG-2 and HuH-7). We found that N1, with an equimolar ratio (CuO: ZnO), was the most potent one that could effectively inhibit cell proliferation, impair cell cycle progression, induce different mechanisms of cell death (apoptosis and autophagy), and suppress HCC migration.

#### The synthesized nanomaterials selectively inhibits growth and survival of HuH-7 cells

To evaluate the inhibitory effects of the N1, N2, and N3 nanocomposites on HCC growth, HepG2, and HuH-7 liver cancer cells were treated with different concentrations for 72 h and assessed using the SRB assay.

**Fig. 3:**
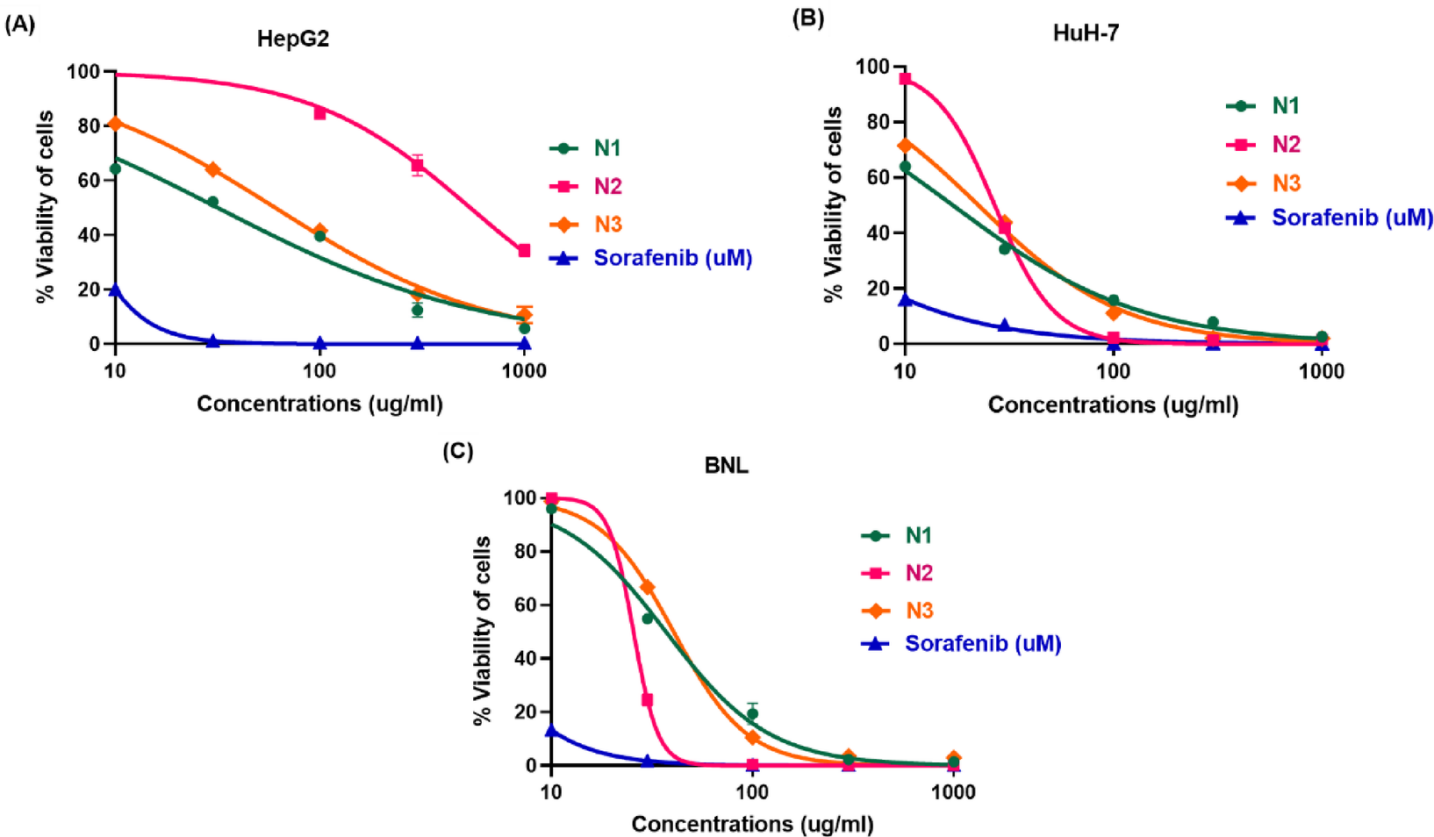
SRB assay evaluating cytotoxic effects of N1, N2, and N3 in (**A**) HepG2, (**B**) HuH-7, and (**C**) BNL cells after treatment for 72 h. Sorafenib was used as a standard. Data are presented as mean ± SD.

As shown in Fig. 3A, N1, and N3 significantly inhibited the proliferation of HepG2 cancer cells in a dose-dependent manner, with IC50 values of 31.69 ± 2.42 µg/mL and 60.58 ± 3.65 µg/mL, respectively, indicating that N1 was more potent in reducing HepG2 cell viability. In contrast, N2 exhibited weak cytotoxic activity against HepG2 cells, with an IC50 value of 543.7 ± 3.91 µg/mL.

Interestingly, HuH-7 cells demonstrated greater sensitivity to all three nanomaterials, as shown in Fig. 3B. N1, N2, and N3 exhibited stronger cytotoxic activity, with IC50 values of 16.99 ± 1.37 µg/mL, 26.93 ± 1.93 µg/mL, and 22.53 ± 1.11 µg/mL, respectively, again highlighting N1 as the most potent nanocomposite in inhibiting HCC cell growth. Therefore, N1 was selected for further analysis of its anticancer effects on the HuH-7 cell line.

Sorafenib, a standard chemotherapy for HCC, demonstrated potent cytotoxicity against both HepG2 (IC50 = 6.09 ± 1.73 µM) and HuH-7 (IC50 = 2.103 ± 0.15 µM) cell lines.

Notably, the cytotoxic effects of N1 and N3 were more pronounced in HuH-7 cancer cells than in normal liver cells (BNL) (Fig. 3C). The IC50 values for N1 and N3 in normal hepatocytes (BNL) were 39.5 ± 2.42 µg/ml and 40.5 ± 2.71 µg/ml, respectively, with selectivity indexes of 2.32 and 1.79, suggesting a relatively favorable safety profile and enhanced selectivity for HuH-7 cells. Conversely, N2 and sorafenib exhibited similar IC50 values in normal (BNL) and HuH-7 cancer cells (25.61 ± 1.82 µg/ml and 1.77 ± 0.96 µM, respectively), implying a lack of selectivity toward cancer cells.

#### N1 arrests HuH-7 cells at S and G2/M phases

**Fig. 4:**
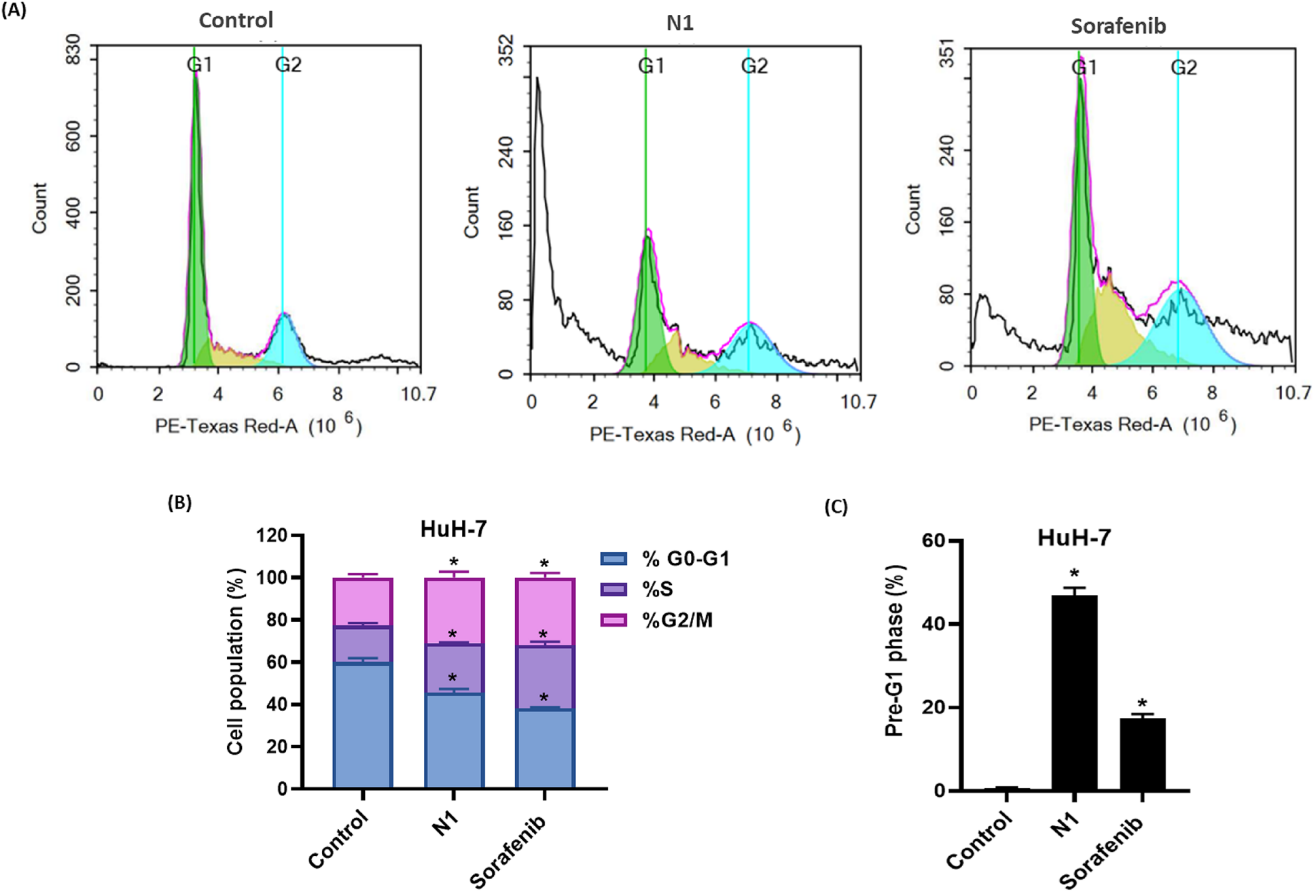
(**A**) Cell cycle distribution in HuH-7 cells after N1 and sorafenib treatment for 48 h was determined using DNA cytometry analysis compared with control (untreated cells). (**B**) The percentage of cells in each phase of the cell cycle was depicted as a bar graph of mean ± SD; *n* = 3. (**C**) The pre-G1 phase was plotted as the percentage of the total cell population. *Statistically significant from control at *p* < 0.05.

To further investigate the antiproliferative and cytotoxic effects of N1 on HuH-7 cells, we performed cell cycle analysis using flow cytometry. The results revealed that, compared to the control, treatment with N1 and sorafenib resulted in a similar pattern of cell cycle distribution but with distinct ratios (Fig. 4A, B).

Treatment with N1 and sorafenib significantly reduced the proliferative fraction in the G0–G1 phase, decreasing it from 60.05 ± 1.96% in untreated control cells to 45.77 ± 1.65% and 38.29 ± 0.45%, respectively. Conversely, both treatments induced an accumulation of cells in the S-phase, increasing from 17.33 ± 1.26% in control cells to 23.23 ± 0.44% (N1) and 29.83 ± 1.58% (sorafenib), suggesting cell cycle arrest and interference with DNA synthesis in liver cancer cells (Fig. 4A, B). This was accompanied by a significant rise in the mitotic G2/M phase population from 22.62 ± 1.78% in control cells to 31 ± 2.82% (N1) and 31.89 ± 2.19% (sorafenib).

These alterations in cell cycle distribution were associated with a robust increase in cell death, as evidenced by a marked elevation in the pre-G1 phase population, rising from 0.78 ± 0.11% in control cells to 46.97 ± 1.78% (N1) and 17.35 ± 1.14% (sorafenib) (Fig. 4C).

### N1 induced apoptotic/necrotic cell death modes of HuH-7 cells

The Annexin V-FITC/PI assay was performed to determine the mechanism of cell death—whether apoptosis or necrosis—in HuH-7 cells following treatment with N1 and sorafenib, particularly after the observed modulation of cell cycle phases, including pre-G1 phase arrest.

**Fig. 5:**
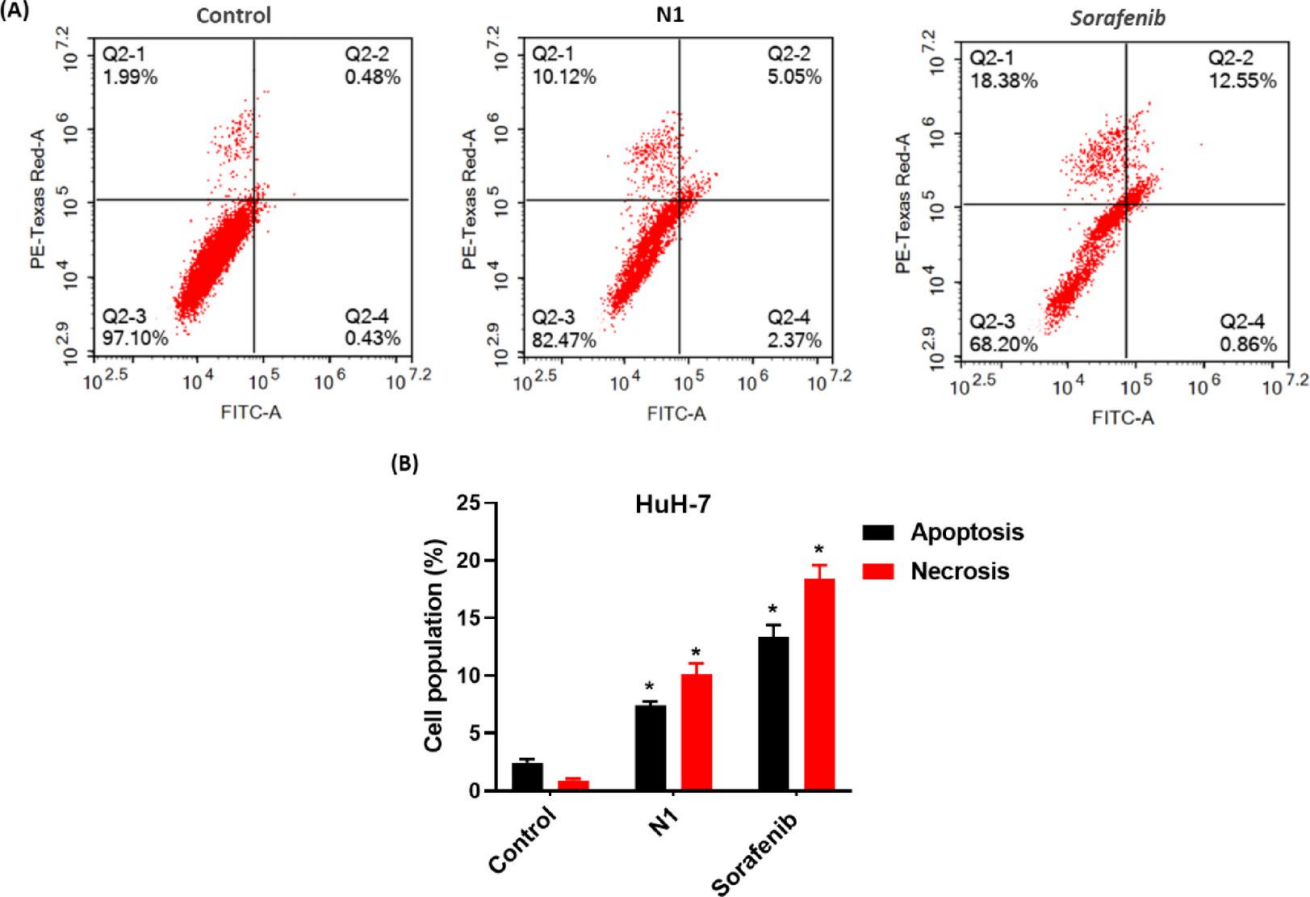
(**A**) Apoptosis/necrosis assessment by flow cytometry in HuH-7 cells pretreated with IC50 values of N1 and sorafenib for 48 h and stained with PI/annexin V-FITC. (**B**) Different cell populations at apoptosis and necrosis were plotted as a percentage of total events. Data are presented as mean ± SD; *n* = 3. *Statistically significant from control at *p* < 0.05. The lower left quadrant (Q3) shows living cells (FITC− and PI−). The lower right quadrant (Q4) represents cells in early apoptosis (FITC+/PI−), and cells in the upper right quadrant (Q2) are cells in late apoptosis (FITC+/PI+). The upper left quadrant (Q1) shows necrotic cells (FITC−/PI+).

As shown in Fig. 5, both N1 and sorafenib induced significant levels of apoptosis and necrosis compared to untreated cells (Fig. 5A, B). Treatment with N1 resulted in approximately 18% total cell death, comprising 7.42 ± 0.33% apoptosis and 10.12 ± 0.96% necrosis. In comparison, sorafenib treatment led to about 32% total cell death, with 13.41 ± 0.99% apoptosis and 18.38 ± 1.22% necrosis. These findings suggest that both treatments exert their potent anticancer effects in HCC by inducing a combination of apoptotic and necrotic cell death.

#### N1 induced autophagy of HuH-7 cells via Beclin-1 upregulation

**Fig. 6:**
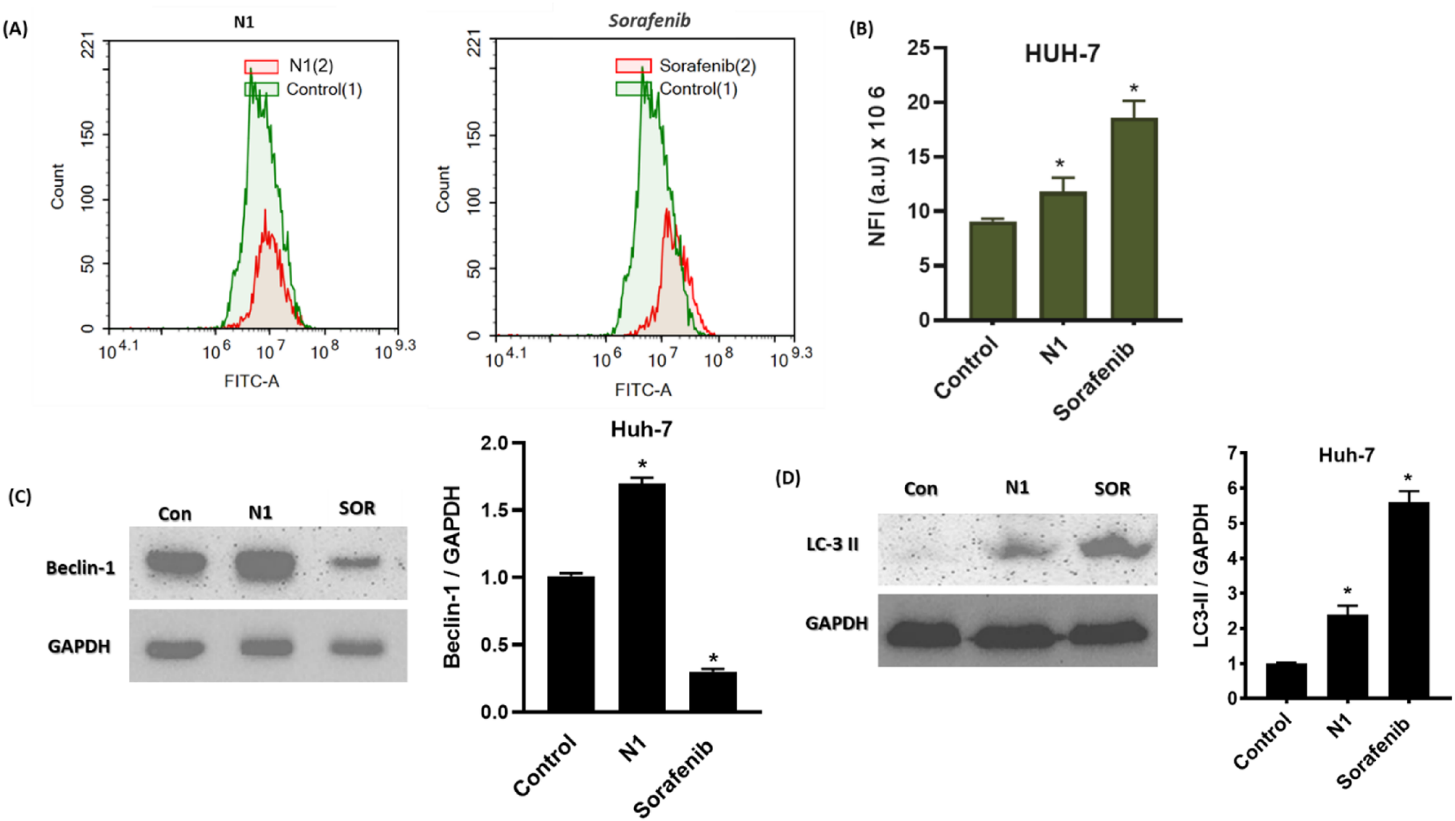
(**A**) Autophagic cell death evaluation in HuH-7 cells after exposure to N1 and sorafenib for 48 h. Cells were stained with acridine orange dye. (**B**) Net fluorescent intensity (NFI) was plotted in comparison with the basal fluorescence of untreated HuH-7 cells. (**C**,**D**) The protein expression of the autophagy markers, Beclin-1 and LC-3 II, was analyzed by Western blotting. Data are displayed in triplicate. *Statistically significant from control at *p* < 0.05.

To investigate whether the observed HuH-7 cell death induced by N1 and sorafenib is associated with autophagy, we assessed autophagic activity after 48 h of treatment using acridine orange staining coupled with flow cytometry (Fig. 6A).

Both N1 and sorafenib treatments significantly increased autophagic cell death, as indicated by a rise in net fluorescent intensity (NFI) from 9.1 ± 0.83 × 10^6^ in control cells to 11.82 ± 1.27 × 10^6^ and 18.59 ± 1.56 × 10^6^, respectively (Fig. 6B). These findings suggest that autophagy contributes to the inhibitory effects of N1 and sorafenib on HCC proliferation and cell survival.

To further confirm this, we examined the expression of the autophagy marker Beclin-1 via Western blot analysis. As shown in Fig. 6C, Beclin-1 expression was significantly upregulated in N1-treated HuH-7 cells. In contrast, sorafenib treatment led to a marked reduction in Beclin-1 levels compared to untreated control cells. These results indicate that while both treatments promote autophagic activity, they may regulate autophagy through distinct molecular mechanisms.

To confirm autophagy induction, the expression of the specific autophagy marker LC3-II was analyzed by Western blot. A significant upregulation of LC3-II expression was observed in HuH-7 cells treated with N1 and sorafenib (Fig. 6D).

#### N1 suppressed the migration of HuH-7 cells

To further explore the anticancer potential of N1, the effects of N1 were compared with sorafenib on HuH-7 cell migration using a scratch wound healing assay. The concentrations used in this assay were significantly lower than the IC_50_ values to prevent cell death or excessive growth inhibition.

**Fig. 7:**
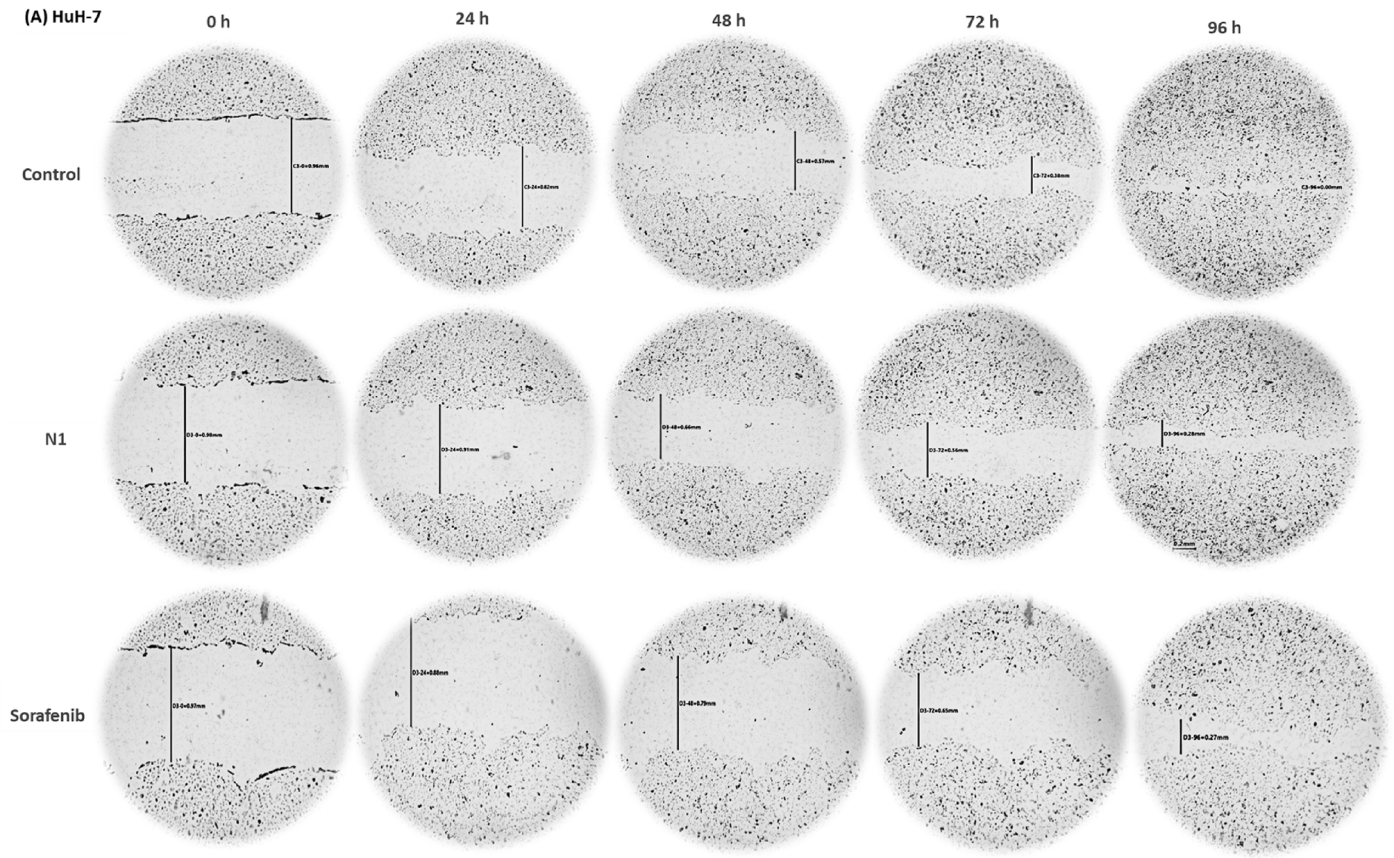

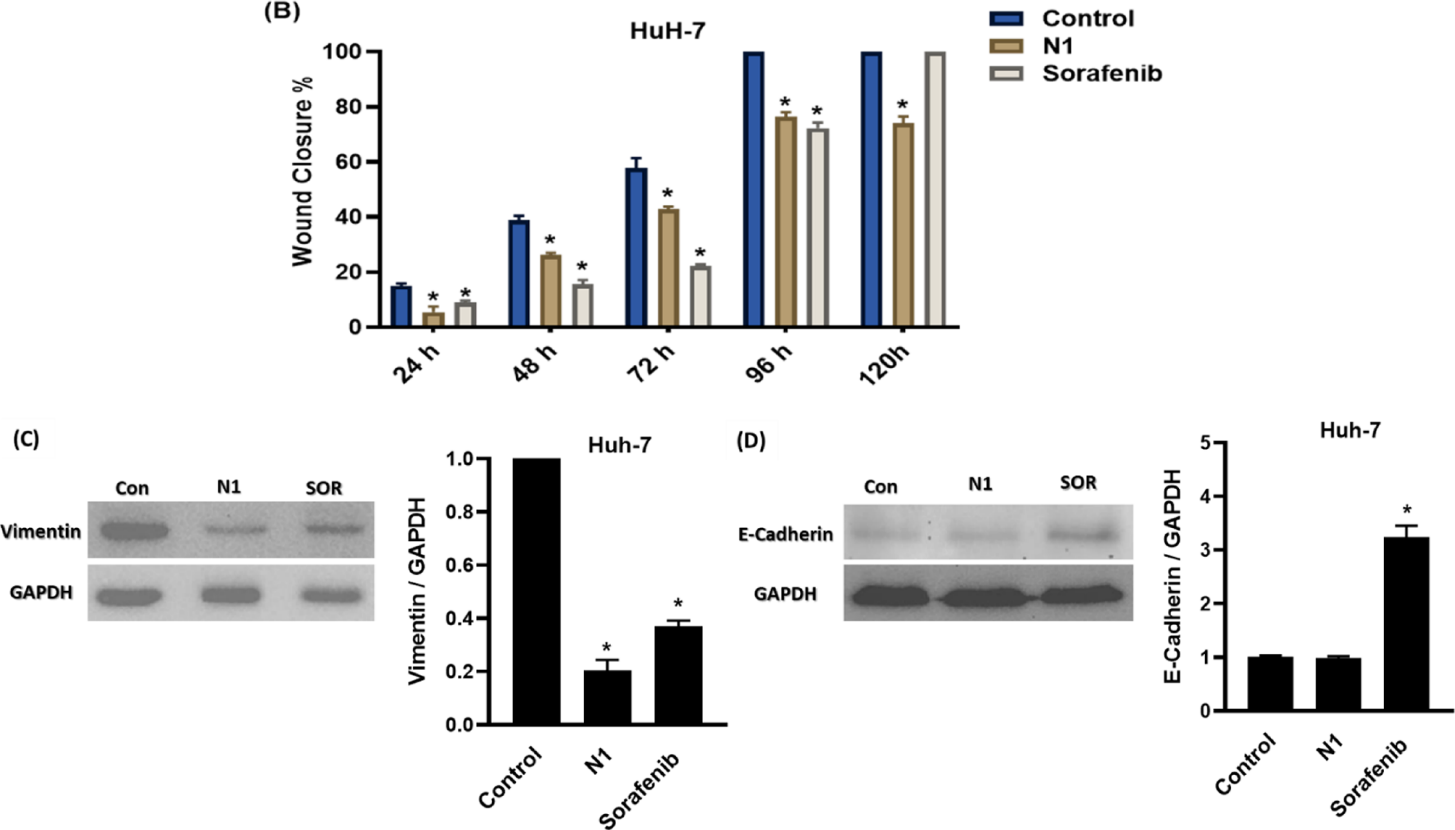
(**A**) Antimigratory activity of N1 and Sorafenib on the migration of HuH-7. The migration widths were measured after treatment daily for 24, 48, 72, 96, and 120 h, respectively. (**B**) Data were blotted as wound closure percent % at each time interval and presented as triplicates. (**C**,**D**) The protein expression of the EMT-related markers, Vimentin and E-cadherin was analyzed by Western blotting. Data are displayed in triplicate. *Statistically significant from control at *p* < 0.05.

At 0 h, the scratch width showed no significant differences among groups (*p* > 0.5), confirming the consistency of the technique. As shown in Fig. 7A, B, untreated HuH-7 cells exhibited a steady migration trend, with scratch closure rates of 15.04 ± 0.79%, 38.81 ± 1.62%, and 57.86 ± 3.60% at 24, 48, and 72 h, respectively, achieving complete closure at 96 h.

Interestingly, N1 (30 µg/ml)—at a concentration lower than the IC_50_—effectively suppressed HuH-7 cell migration at all tested time points (24, 48, 72, 96, and 120 h). The scratch closure percentages were significantly reduced to 5.51 ± 1.06%, 26.16 ± 0.89%, 42.9 ± 2.85%, 76.45 ± 1.69%, and 74.06 ± 2.45%, respectively, with no complete closure even after 120 h.

Similarly, sorafenib inhibited cell migration but to a lesser extent than N1. The scratch closure percentages for sorafenib-treated cells were 8.96 ± 0.62%, 15.55 ± 1.57%, 22.27 ± 0.56%, and 72.29 ± 2.01% at 24, 48, 72, and 96 h, respectively, with complete closure observed at 120 h (Fig. 7A, B).

These findings highlight N1 as a promising antimetastatic agent in HCC, demonstrating superior efficacy compared to sorafenib. To further assess the anti-migratory effects of N1 and sorafenib, western blot analysis was performed to evaluate vimentin, a key protein in the EMT pathway. As shown in Fig. 7C, both treatments significantly downregulated vimentin expression compared to the control, with N1 exhibiting a stronger inhibitory effect than sorafenib.

To further support these findings, E-cadherin expression (a well-known marker of EMT), that the loss of its expression occurs during tumor cell progression and metastasis, was analyzed by Western blot. Sorafenib significantly increased its expression in treated HuH-7 cells, whereas N1 did not show a noticeable change compared to untreated HuH-7 cells (Fig. 7D).

#### N1 induced ROS generation in HuH-7 cells

**Fig. 8:**
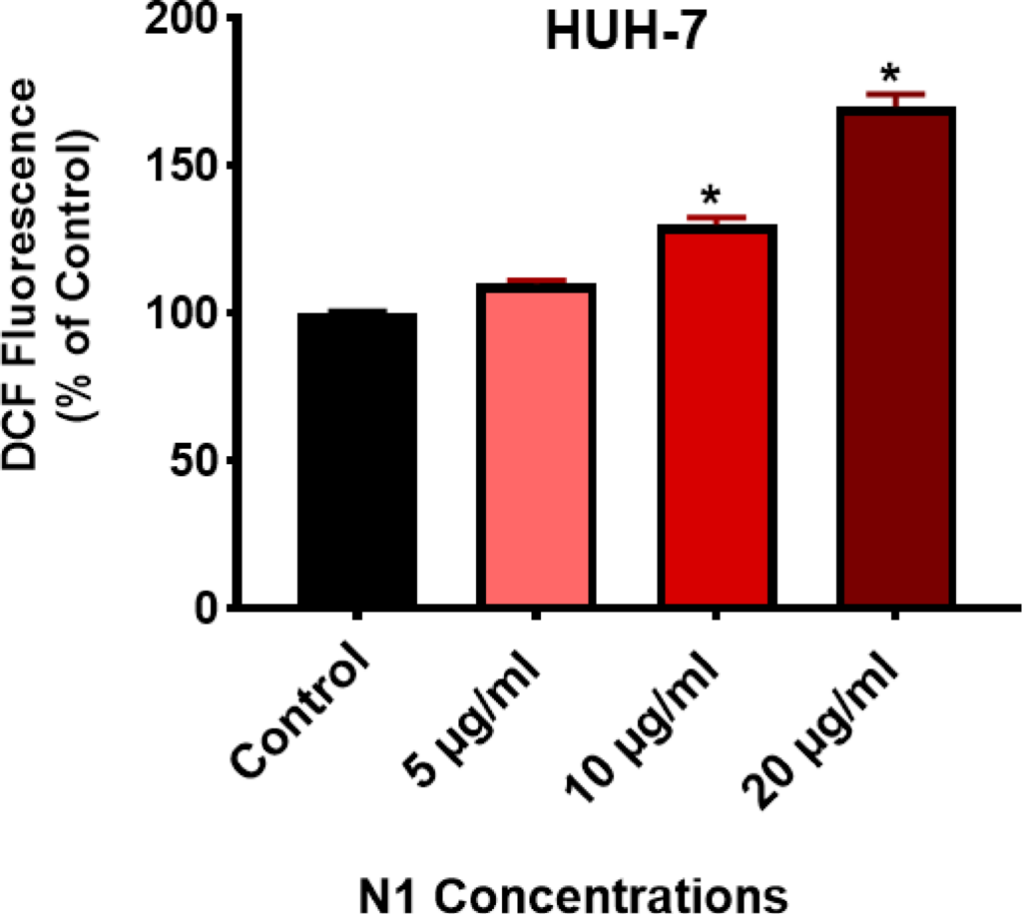
Induced ROS production by N1 nanocomposite in HuH-7 cells. Results are illustrated as a percentage relative to the untreated control cells after treatment with 5,10, and 20 µg/ml of nanocomposite for 24 h. Data are displayed in triplicate. *Statistically significant from control at *p* < 0.05.

To verify the role of ROS generation in ZnO/CuO nanocomposite (N1)-mediated anticancer activity and apoptosis induction, HuH-7 cells were treated with 5, 10, and 20 µg/mL of N1 for 24 h and ROS levels were assessed (Fig. 8). Our data indicated that the synthesized nanocomposite N1 significantly (*P* < 0.05) induced the production of ROS in HuH-7 cells in a dose-dependent manner compared to the untreated cells, suggesting that anticancer activity of ZnO/CuO nanocomposite may involve ROS-mediated apoptosis in liver cancer cells.

## Discussion

Hepatocellular carcinoma, a highly prevalent and lethal malignancy, presents a major clinical challenge due to the limited efficacy of existing treatment strategies. Despite significant therapeutic advancements, the 5-year survival rate remains critically low, primarily due to chemotherapy-induced adverse effects, including cachexia and drug resistance, as well as the failure to effectively suppress tumor invasion and metastasis^47^.

The in vitro anticancer potential of ZnO/CuO-NPs against the HuH-7 cell line was evaluated in comparison to sorafenib, the first approved drug for the treatment of advanced hepatocellular carcinoma^48^. Sorafenib treatment effectively inhibited HCC growth by inducing cell cycle arrest, apoptosis, and necrosis, consistent with previous reports^6,49^.

Nanocomposites have been observed to play an integral role in signaling cascades, which makes it imperative to identify them as therapeutic targets for cancer. Numerous studies have investigated the improved anticancer efficacies of ZnO/CuO-NPs against various cancer types, such as breast cancer^50^, colorectal cancer^51^, lung cancer^52^, and ovarian cancer^53^. The cytotoxic efficacy of these NPs correlates with NP composition, concentration, size, shape, surface charge, surface area, and dispersion states^54^.

According to the cytotoxicity data, there is a difference in the IC50 values of three synthesized nanocomposites with varying ratios of CuO-NPs and ZnO-NPs. N1 and N3 nanocomposites, with higher CuO ratios (50% and 85%), exhibited greater cytotoxicity against HCC cell lines (HepG2 and HuH-7) than N2 nanocomposite, which contained a lower CuO content (15%). Notably, N1, composed of 50% CuO and 50% ZnO, demonstrated the most potent effect, suggesting a synergistic interaction between both metal oxide nanoparticles. Additionally, the three tested nanocomposites displayed significantly higher cytotoxic activity against the HuH-7 cell line compared to the HepG2 cell line, confirming that the cytotoxicity of these nanoparticles varies among different cell lines^55^, even if from the same source.

Despite the potent cytotoxic activity of sorafenib, its IC50 values for cancer and normal cell lines are approximately similar, emphasizing the need for safer treatment alternatives for HCC. Evaluating cytotoxicity on normal (non-cancerous) human cell lines is considered a critical initial step in assessing the safety of synthesized nanocomposites^56^. In contrast, nanocomposites exhibited significantly lower cytotoxicity against normal BNL cells compared to sorafenib. Among the tested nanocomposites, N1 and N3 displayed comparable IC50 values against BNL normal liver cells, with selectivity indexes of 2.32 and 1.79, respectively. These findings align with previous reports on the selective toxicity of ZnO nanoparticles (ZnO-NPs) toward cancer cells^57^, and increasing its ratio in the nanoparticles boosted selectivity and confirmed the biocompatibility of these nanocomposites with normal cells since ZnO-NPs are relatively affordable and less toxic than other metal oxide NPs^58^. This verifies previous research findings showing that while CuO-NPs show potent cytotoxicity in cancer cells, they are harmful in healthy tissues^59^. Several studies suggested that the safety of CuO-NPs is related to the source of NPS, with biogenic CuO-NPs having higher relative safety for normal human cell lines than biosynthesized CuO-NPs, implying that green-synthesized CuO-NPs are required for improved safety^15^. Additionally, the mechanism by which these NPs exert anticancer effects may vary depending on their synthesis method^60,61^.

Nanocomposites strongly affect crucial cellular outcomes, such as cell cycle and proliferation, and can trigger different modes of cell death. These effects depend on their physicochemical properties comprising shape, dispersity state, charge, and size^14,62^. In our work, treating HuH-7 cells with ZnO/CuO nanocomposites stops both the DNA synthesis as well as the cell division by arresting the cells in S-phase and G2-M phase, respectively, thus enhancing the process of cell death reflected in the sub-G1 phase. This is in line with previous studies implying cell cycle arrest at these phases in the observed inhibition of ZnO-NPs and/or CuO-NPs of cancer cells^26,63^.

The present research revealed that ZnO and CuO nanocomposites influence multiple cellular processes, including apoptosis, necrosis, and autophagy. Apoptosis plays a crucial role in cancer development and progression, as one of the hallmark characteristics of cancer is its ability to evade apoptotic cell death and sustain uncontrolled proliferation. Consequently, targeting apoptosis remains a key strategy in cancer treatment, and it represents one of the primary mechanisms through which anticancer agents exert cytotoxic effects^64^. NPs have been shown to trigger cell apoptosis as their principal mechanism of killing cancer cells^65,66^.

Annexin V-FITC/PI staining was performed to distinguish apoptotic and necrotic cell populations^67^ in HuH-7 cells following treatment with N1 and sorafenib. In the current study, both treatments significantly increased apoptotic and necrotic cell populations compared to untreated controls. The observed apoptosis aligns with previous studies demonstrating the role of mitochondrial-mediated apoptosis as a critical therapeutic target in cancer treatment^68^. Mitochondrial dysfunction plays a pivotal role in nanoparticle-induced apoptosis, as nanoparticles have been reported to enhance ROS production, cytochrome c release, and caspase activation, leading to programmed cell death. Similar apoptotic mechanisms have been observed in other metal-based nanoparticles, where mitochondrial membrane depolarization triggers cell death pathways^69^.

Similarly, Kumar et al. (2024) demonstrated that fluoronucleoside, a fluorinated nucleoside compound, effectively inhibits non-Hodgkin lymphoma through ROS generation, cell cycle arrest, and apoptosis, further supporting the notion that targeting apoptosis remains a fundamental approach in cancer therapy^68^. It has also emphasized the role of apoptosis in fluoronucleoside-induced inhibition of Dalton’s lymphoma growth and proliferation, highlighting the therapeutic significance of activating intrinsic apoptotic pathways in cancer treatment^70^.

These findings align with the current study, as ZnO/CuO nanocomposites significantly triggered apoptotic pathways in HuH-7 cells, increased the intracellular levels of ROS that reinforcing their therapeutic potential against HCC. The ability of these nanocomposites to induce apoptosis further underscores their promise as effective anticancer agents, paralleling recent advances in targeted cancer therapies. The ability of N1 and sorafenib to induce apoptosis and necrosis further supports their potential as effective anticancer agents in HCC therapy. Oxidative stress plays a crucial role in the cytotoxic effects of metal oxide nanoparticles, further supporting the potential of ZnO/CuO nanocomposite as an effective therapeutic agent targeting ROS-mediated apoptosis in liver cancer cells.

Over the years, the role of autophagy on tumors, including HCC, has been complex and multifaceted, acting as a double-edged sword in tumorigenesis, metastasis, and therapeutic resistance^71^. Meanwhile, excessive autophagy induction can also inhibit tumor cell growth and proliferation and even lead to cell death^72^. Several studies have reported that numerous metal oxide NPs display anticancer activity by inducing autophagy^73–75^.

Moreover, many antitumor agents can inhibit cell migration by inducing cancer cell autophagy^76^. Therefore, modulation of autophagy has been considered a promising strategy for cancer therapy^77^. Several studies have demonstrated that nanomaterials significantly impact cancer cell migration pathways, often by modulating key molecular targets involved in EMT^78^. The current study demonstrated that ZnO/CuO nanocomposite upregulated beclin-1 protein expression in HCC cells. Further research found that increasing the beclin-1 expression leads to inducing cell autophagy^2,5^. Additionally, the ability of these nanocomposites to modulate intracellular signaling pathways involved in migration, further highlights their potential as therapeutic agents in limiting cancer metastasis.

Interestingly, ZnO/CuO nanocomposite did not significantly alter E-cadherin expression, one of key EMT markers that known to be downregulated during cancer progression and metastasis^79^. Given its perceptible low expression in the studied cell line, this suggests that the antimigratory activity of ZnO/ CuO nanocomposite may not be attributed to a modulatory effect on E-cadherin expression.

Importantly, autophagy has been recognized as a viable therapeutic target in multiple cancers, including HCC, where promoting autophagic cell death may enhance anticancer efficacy. In line with this, Fayzullina et al. (2022) highlighted autophagy as a potential therapeutic strategy in Ewing sarcoma, demonstrating that targeted autophagy modulation can sensitize tumor cells to treatment and enhance therapeutic outcomes^80^. Their findings suggest that regulating autophagy pathways could be instrumental in overcoming drug resistance and improving treatment efficacy in aggressive malignancies. These insights further support our results, indicating that ZnO/CuO nanocomposites may exert their anticancer effects not only through apoptosis and necrosis but also via autophagic cell death, positioning autophagy as a crucial therapeutic mechanism in HCC treatment.

Thus, this study underscores the therapeutic potential of autophagy modulation in HCC and highlights ZnO/CuO nanocomposites as a promising strategy for targeting this pathway. However, further mechanistic investigations are necessary to fully elucidate the interplay between autophagy, apoptosis, and necrosis in the anticancer effects of these nanocomposites and to optimize their clinical applicability. In the context of HCC, promoting tumor necrosis has also been identified as an underlying mechanism of successful therapy^81^, and in NP-induced cell death^82^.

Autophagy and apoptosis pathways can be independent or parallel, and one may influence the other^83^. It has been shown that triggering both apoptotic and autophagic cell death pathways shows promise in decreasing tumor growth in HCC and could be an intriguing option for HCC therapy^84^. Nanocomposites, such as ZnO and CuO, have been shown to promote both apoptosis and autophagy as the primary mechanisms underlying the anticancer properties in many cancer cell lines^85,86^.

More than 90% of HCC-related deaths resulted from metastasis, one of the most vital mechanisms driving HCC progression^87^. Effective treatment strategies must target fundamental factors involved in metastasis, such as EMT, which plays an essential role in the early phases of metastasis^88^, and its inhibition is critical for improving patient survival.

Epithelial–mesenchymal transition is a fundamental biological process through which cancer cells acquire migratory and invasive properties, enabling tumor progression and metastasis^89^. This process involves epithelial cells losing their cell polarity and cell-to-cell adhesion and converting into a mesenchymal phenotype with migratory and invasive properties marked by increased vimentin levels^90,91^. Moreover, the elevated expression level of vimentin in HCC patients is intensely correlated with poor tumor differentiation, vascular invasion, extrahepatic recurrence, and reduced disease-free survival post-surgery^8,92^.

Nanocomposites demonstrated significant inhibition of HCC cell migration, as evidenced by the migration assay. This effect was further supported by Western blot analysis, which revealed a marked downregulation of vimentin protein expression—a key regulator of EMT associated with cancer cell motility and invasiveness. These findings underscore the potential of CuO and ZnO-based nanocomposites as promising therapeutic agents for HCC by targeting critical pathways involved in tumor progression. The compelling anti-migratory effects observed emphasize the necessity for further investigation and clinical translation to develop more effective and selective HCC treatment strategies.

## Conclusion

This study provides compelling evidence for the anticancer potential of bimetallic ZnO/CuO nanocomposites against hepatocellular carcinoma (HCC). Our findings demonstrate that these nanocomposites significantly inhibited HuH-7 cell proliferation, induced cell cycle arrest at the S and G2/M phases, and triggered multiple cell death pathways, including apoptosis, necrosis, and autophagy. Notably, ZnO/CuO nanocomposites effectively suppressed cell migration by downregulating vimentin, a key EMT marker, emphasizing their role in preventing metastasis.

The chemical characterization techniques, including XPS and X-ray diffraction, provided a comprehensive understanding of the synthesized nanocomposites, confirming their crystal structure, particle size, and oxidation states. These analyses validated their physicochemical properties, which contributed to their potent anticancer activity. Importantly, ZnO/CuO nanocomposites exhibited selective cytotoxicity toward cancer cells while demonstrating reduced toxicity to normal liver cells, suggesting their potential as a safer alternative to sorafenib.

Overall, this study highlights the therapeutic promise of ZnO/CuO nanocomposites as a novel anticancer strategy for HCC treatment. Further in vivo and mechanistic studies are warranted to explore their full clinical potential and optimize their biocompatibility for future therapeutic applications.

## Future directions

Future studies should further explore the mechanistic pathways underlying the anticancer effects of ZnO/CuO nanocomposites, particularly their role in mitochondrial membrane potential (MMP) disruption and ROS-mediated apoptosis. Mitochondrial dysfunction, cytochrome c release, and caspase activation are key processes in apoptosis that warrant detailed investigation in this context^70,93^. Additionally, the long-term impact of these nanocomposites on tumor progression and metastasis should be assessed through in vitro and in vivo models. Exploring their combinatory effects with existing chemotherapeutic agents could further enhance their efficacy while minimizing toxicity. Expanding on these insights will help optimize the therapeutic potential of ZnO/CuO nanocomposites in HCC treatment.

## Electronic supplementary material

Below is the link to the electronic supplementary material.

Supplementary Material 1

## Data Availability

Data is provided within the manuscript or supplementary information files.

## References

[1] Bray, F. et al. Global cancer statistics 2022: GLOBOCAN estimates of incidence and mortality worldwide for 36 cancers in 185 countries. *CA Cancer J. Clin.* **74**, 229–263 (2024). 38572751 10.3322/caac.21834

[2] Liao, Y. et al. Animal-derived natural products for hepatocellular carcinoma therapy: Current evidence and future perspectives. *Front. Pharmacol.* **15**, 1–18 (2024). 10.3389/fphar.2024.1399882 PMC11129636 38803433

[3] Huang, X. et al. Advances and applications of nanoparticles in cancer therapy. *MedComm Oncol.*** 3** (2024).

[4] Guo, J. et al. Therapeutic effects of natural products on liver cancer and their potential mechanisms. *Nutrients* **16**, 1642 (2024). 38892575 10.3390/nu16111642 PMC11174683

[5] Chen, S. et al. Loss of SPTBN1 suppresses autophagy via SETD7-mediated YAP methylation in hepatocellular carcinoma initiation and development. *CMGH* **13**, 949–973e7 (2022). 34737104 10.1016/j.jcmgh.2021.10.012 PMC8864474

[6] Sethi, G. et al. Apoptotic mechanisms of Quercetin in liver cancer: Recent trends and advancements. *Pharmaceutics* **15**, 1–20 (2023). 10.3390/pharmaceutics15020712 PMC9960374 36840034

[7] Li, Y., Yin, Y., He, Y., He, K. & Li, J. SOS1 regulates HCC cell epithelial–mesenchymal transition via the PI3K/AKT/mTOR pathway. *Biochem. Biophys. Res. Commun.* **637**, 161–169 (2022). 36403479 10.1016/j.bbrc.2022.11.015

[8] Du, Y. Q. et al. Plumbagin regulates snail to inhibit hepatocellular carcinoma epithelial–mesenchymal transition in vivo and in vitro. *J. Hepatocell. Carcinoma* **11**, 565–580 (2024). 38525157 10.2147/JHC.S452924 PMC10960549

[9] Seydi, H. et al. Autophagy orchestrates resistance in hepatocellular carcinoma cells. *Biomed. Pharmacother*. **161**, 114487 (2023). 36963361 10.1016/j.biopha.2023.114487

[10] Huang, A., Yang, X. R., Chung, W. Y., Dennison, A. R. & Zhou, J. Targeted therapy for hepatocellular carcinoma. *Signal. Transduct. Target. Ther.* **5**, 146 (2020). 32782275 10.1038/s41392-020-00264-x PMC7419547

[11] Olarewaju, O. et al. MicroRNA miR-20a-5p targets CYCS to inhibit apoptosis in hepatocellular carcinoma. *Cell. Death Dis.* **15** (2024). 10.1038/s41419-024-06841-0 PMC11211328 38937450

[12] Sedighi, M. et al. Nanomedicines for hepatocellular carcinoma therapy: Challenges and clinical applications. *Mater. Today Commun.* **34**, 105242 (2023).

[13] Anjum, S. et al. Recent advances in zinc oxide nanoparticles (Zno nps) for cancer diagnosis, target drug delivery, and treatment. *Cancers (Basel)*** 13** (2021). 10.3390/cancers13184570 PMC8468934 34572797

[14] Mitchell, M. J. et al. Engineering precision nanoparticles for drug delivery. *Nat. Rev. Drug Discov*. **20**, 101–124 (2021). 33277608 10.1038/s41573-020-0090-8 PMC7717100

[15] Gebreslassie, Y. T. & Gebremeskel, F. G. Green and cost-effective biofabrication of copper oxide nanoparticles: Exploring antimicrobial and anticancer applications. *Biotechnol. Rep.* **41**, e00828 (2024). 10.1016/j.btre.2024.e00828 PMC10835232 38312482

[16] Ferrone, E., Araneo, R., Notargiacomo, A., Pea, M. & Rinaldi, A. ZnO nanostructures and electrospun ZnO–polymeric hybrid nanomaterials in biomedical, health, and sustainability applications. *Nanomaterials* **9**, 1449 (2019). 31614707 10.3390/nano9101449 PMC6835458

[17] Rodríguez-Barajas, N. et al. Study of the interaction of Ti–Zn as a mixed oxide at different pH values synthesized by the sol–gel method and its antibacterial properties. *Nanomaterials* **12**, 1948 (2022). 35745287 10.3390/nano12121948 PMC9229482

[18] Jiang, J., Pi, J. & Cai, J. The advancing of zinc oxide nanoparticles for biomedical applications. *Bioinorg. Chem. Appl.* **2018**, 1–18 (2018). 10.1155/2018/1062562 PMC6057429 30073019

[19] Abbasi, B. A. et al. Bioactivities of *Geranium wallichianum* leaf extracts conjugated with zinc oxide nanoparticles. *Biomolecules* **10**, 38 (2019). 31888037 10.3390/biom10010038 PMC7022592

[20] Qamar, H., Rehman, S., Chauhan, D. K., Tiwari, A. K. & Upmanyu, V. Green synthesis, characterization and antimicrobial activity of copper oxide nanomaterial derived from *Momordica charantia*. *Int. J. Nanomed.* **15**, 2541–2553 (2020). 10.2147/IJN.S240232 PMC7170629 32368039

[21] Sarfraz, M. H. et al. Comparative analysis of phyto-fabricated Chitosan, copper oxide, and chitosan-based CuO nanoparticles: Antibacterial potential against *Acinetobacter baumannii* isolates and anticancer activity against HepG2 cell lines. *Front. Microbiol.* **14** (2023). 10.3389/fmicb.2023.1188743 PMC10264586 37323910

[22] Singh, D. et al. Bacteria assisted green synthesis of copper oxide nanoparticles and their potential applications as antimicrobial agents and plant growth stimulants. *Front. Chem.* **11** (2023). 10.3389/fchem.2023.1154128 PMC10119401 37090246

[23] Chang, Y. N., Zhang, M., Xia, L., Zhang, J. & Xing, G. The toxic effects and mechanisms of CuO and ZnO nanoparticles. *Materials (Basel)* **5**, 2850–2871 (2012).

[24] Daimari, J. & Deka, A. K. Anticancer, antimicrobial and antioxidant activity of CuO–ZnO bimetallic nanoparticles: Green synthesised from *Eryngium foetidum* leaf extract. *Sci. Rep.* **14**, 1–14 (2024). 39174638 10.1038/s41598-024-69847-w PMC11341821

[25] Alijani, H. Q. et al. Biosynthesis of spinel nickel ferrite nanowhiskers and their biomedical applications. *Sci. Rep.* **11**, 17431 (2021). 34465814 10.1038/s41598-021-96918-z PMC8408215

[26] Cao, Y. et al. Green synthesis of bimetallic ZnO–CuO nanoparticles and their cytotoxicity properties. *Sci. Rep.* **11**, 23479 (2021). 34873281 10.1038/s41598-021-02937-1 PMC8648779

[27] Nguyen, P. H. D., Jayasinghe, M. K., Le, A. H., Peng, B. & Le, M. T. N. Advances in drug delivery systems based on red blood cells and their membrane-derived nanoparticles. *ACS Nano* **17**, 5187–5210 (2023). 36896898 10.1021/acsnano.2c11965

[28] Himoto, T. & Masaki, T. Current trends on the involvement of zinc, copper, and selenium in the process of hepatocarcinogenesis. *Nutrients* **16**, 1–21 (2024). 10.3390/nu16040472 PMC10892613 38398797

[29] El-Seidy, A. M. A., Elbaset, M. A., Ibrahim, F. A. A., Moussa, A., Bashandy, S. A. & S. A. & Nano cerium oxide and cerium/zinc nanocomposites characterization and therapeutic role in combating obesity via controlling oxidative stress and insulin resistance in rat model. *J. Trace Elem. Med. Biol.* **80**, 127312 (2023). 37804595 10.1016/j.jtemb.2023.127312

[30] Bashandy, S. A. E. et al. Zinc nanoparticles ameliorated obesity-induced cardiovascular disease: Role of metabolic syndrome and iron overload. *Sci. Rep.* **13**, 16010 (2023). 37749096 10.1038/s41598-023-42550-y PMC10519991

[31] Elseidy, A. et al. Zinc oxide nanoparticles characterization and therapeutic evaluation on high fat/sucrose diet induced-obesity. *Egypt. J. Chem.*10.21608/ejchem.2022.112166.5113 (2022).

[32] Abdel-Sattar, O. E. et al. Cytotoxic and chemomodulatory effects of *Phyllanthus niruri* in MCF-7 and MCF-7ADR breast cancer cells. *Sci. Rep.* **13**, 2683 (2023). 36792619 10.1038/s41598-023-29566-0 PMC9932073

[33] Abdel-Sattar, O. E. et al. Hypophyllanthin and phyllanthin from *Phyllanthus niruri* synergize doxorubicin anticancer properties against resistant breast cancer cells. *ACS Omega* **8**, 28563–28576 (2023). 37576627 10.1021/acsomega.3c02953 PMC10413485

[34] Shari, K. et al. Jatrophone: A cytotoxic macrocylic diterpene targeting PI3K/AKT/NF-κB pathway, inducing apoptosis and autophagy in resistant breast cancer cells. *BMC Complement. Med. Ther.* **23**, 293 (2023). 37608270 10.1186/s12906-023-04113-6 PMC10463460

[35] Mokbel, H. A. Q. et al. Evaluation of in-vitro anticancer activity of *Vernonia leopoldii* (Sch. Blip.) methanolic extract on HepG2 human cancer cells, relative to its phytochemical contents determined by LC-MS/MS fingerprint. *Egypt. J. Chem.*10.21608/ejchem.2024.309438.10138 (2024).

[36] Hassabo, A. A., Abdelraof, M. & Allam, R. M. L-arginase from *Streptomyces diastaticus* MAM5 as a potential therapeutic agent in breast cancer: Purification, characterization, G1 phase arrest and autophagy induction. *Int. J. Biol. Macromol.*. 10.1016/j.ijbiomac.2022.10.152 (2022). 10.1016/j.ijbiomac.2022.10.152 36302487

[37] Chen, Y., Chen, X., Ding, X., Wang, Y. & Afatinib An EGFR inhibitor, decreases EMT and tumorigenesis of Huh-7 cells by regulating the ERK-VEGF/MMP9 signaling pathway. *Mol. Med. Rep.* **20**, 3317–3325 (2019). 31432165 10.3892/mmr.2019.10562 PMC6755195

[38] Sarabikia, H., Souri, R. & Safaei, M. Evaluating the anticancer effects of polyvinyl alcohol/magnesium oxide Bionanocomposite on human oral cancer cells. *J. Kermanshah Univ. Med. Sci.* **27** (2023).

[39] Nili, H. et al. Alkali ratio control for lead-free piezoelectric thin films utilizing elemental diffusivities in RF plasma. *CrystEngComm* **15**, 7222 (2013).

[40] Torregrosa-Rivero, V., Moreno-Marcos, C., Albaladejo-Fuentes, V., Sánchez-Adsuar, M. S. & Illán-Gómez, M.-J. BaFe1–xCuxO3 perovskites as active phase for diesel (DPF) and gasoline particle filters (GPF). *Nanomaterials* **9**, 1551 (2019). 31683700 10.3390/nano9111551 PMC6915380

[41] Xu, L., Zhang, D., Ming, L., Jiao, Y. & Chen, F. Synergistic effect of interfacial lattice Ag++ and Ag+ 0 clusters in enhancing the photocatalytic performance of TiO2. *Phys. Chem. Chem. Phys.* **16**, 19358 (2014). 25099521 10.1039/c4cp02658f

[42] Ateia, E. E., Arman, M. M. & Mohamed, A. T. A facile novel synthesis of AgCuO2 delafossite nanoparticles and evaluation of their antimicrobial activity. *Sci. Rep.* **13**, 3141 (2023). 36823448 10.1038/s41598-023-30255-1 PMC9950047

[43] Qiu, J. et al. Catalytic activity, selectivity, and stability of co-precipitation synthesized Mn-Ce mixed oxides for the oxidation of 1,2-dichlorobenzene. *Environ. Sci. Pollut. Res.* **28**, 65416–65427 (2021). 10.1007/s11356-021-15016-9 34319524

[44] El-Okaily, M. S. et al. Nanoarchitectonics of catalytic tubular nanomotors based on Cu/Fe@SBA-15 for lung cancer treatment. *J. Mater. Res.* **39**, 1741–1757 (2024).

[45] Si, D., Xiong, B., Chen, L. & Shi, J. Highly selective and efficient electrocatalytic synthesis of glycolic acid in coupling with hydrogen evolution. *Chem. Catal.* **1**, 941–955 (2021).

[46] Miura, R., Kitada, A., Fukami, K. & Murase, K. Thermodynamic design of electrolyte for CuO/Cu2O bilayer by anodic electrodeposition. *J. Electrochem. Soc.* **168**, 062506 (2021).

[47] Zheng, S. et al. Hepatocellular carcinoma: Current drug therapeutic status, advances and challenges. *Cancers (Basel)*. **16**, 1582 (2024). 38672664 10.3390/cancers16081582 PMC11048862

[48] Mousa, A. Sorafenib in the treatment of advanced hepatocellular carcinoma. *Saudi J. Gastroenterol.* **14**, 40 (2008). 19568496 10.4103/1319-3767.37808 PMC2702892

[49] Abdu, S., Juaid, N., Amin, A., Moulay, M. & Miled, N. Effects of sorafenib and quercetin alone or in combination in treating hepatocellular carcinoma: In vitro and in vivo approaches. *Molecules* **27**, 8082 (2022). 10.3390/molecules27228082 PMC9697794 36432184

[50] Dolati, M., Tafvizi, F., Salehipour, M., Komeili Movahed, T. & Jafari, P. Biogenic copper oxide nanoparticles from *Bacillus coagulans* induced reactive oxygen species generation and apoptotic and anti-metastatic activities in breast cancer cells. *Sci. Rep.* **13**, 3256 (2023). 36828883 10.1038/s41598-023-30436-y PMC9958044

[51] Efati, Z. et al. Green chemistry synthesized zinc oxide nanoparticles in *Lepidium sativum* L. seed extract and evaluation of their anticancer activity in human colorectal cancer cells. *Ceram. Int.* **49**, 32568–32576 (2023).

[52] Rani, N. et al. Azadirachta indica leaf extract mediated biosynthesized rod-shaped zinc oxide nanoparticles for in vitro lung cancer treatment. *Mater. Sci. Eng. B*. **284**, 115851 (2022).

[53] Mousa, A. B., Moawad, R., Abdallah, Y., Abdel-Rasheed, M. & Zaher, A. M. A. Zinc oxide nanoparticles promise anticancer and antibacterial activity in ovarian cancer. *Pharm. Res.* **40**, 2281–2290 (2023). 37016170 10.1007/s11095-023-03505-0 PMC10072921

[54] Naser, S. S. et al. Emerging trends in the application of green synthesized biocompatible ZnO nanoparticles for translational paradigm in cancer therapy. *J. Nanotheranostics*. **4**, 248–279 (2023).

[55] Sahu, D., Kannan, G. M., Tailang, M. & Vijayaraghavan, R. In vitro cytotoxicity of nanoparticles: A comparison between particle size and cell type. *J. Nanosci.* 1–9 (2016).

[56] Orshiso, T. A. et al. One-pot biopreparation of trimetallic ZnO–MgO–CuO nanoparticles: Enhanced cytotoxicity, antibacterial activities and molecular Docking studies. *Chem. Afr.* **7**, 1963–1980 (2024).

[57] Hadi, A. J., Nayef, U. M., Mutlak, F. A. H. & Jabir, M. S. Laser-ablated zinc oxide nanoparticles and evaluation of their antibacterial and anticancer activity against an ovarian cancer cell line: In vitro study. *Plasmonics* **18**, 2091–2101 (2023).

[58] Mishra, P. K., Mishra, H., Ekielski, A., Talegaonkar, S. & Vaidya, B. Zinc oxide nanoparticles: A promising nanomaterial for biomedical applications. *Drug Discov. Today*. **22**, 1825–1834 (2017). 28847758 10.1016/j.drudis.2017.08.006

[59] Naz, S., Gul, A. & Zia, M. Toxicity of copper oxide nanoparticles: A review study. *IET Nanobiotechnol.* **14**, 1–13 (2020). 31935671 10.1049/iet-nbt.2019.0176 PMC8676634

[60] Murali, M. et al. Plant-mediated zinc oxide nanoparticles: Advances in the new millennium towards understanding their therapeutic role in biomedical applications. *Pharmaceutics* **13**, 1662 (2021). 34683954 10.3390/pharmaceutics13101662 PMC8540056

[61] Rodríguez-Barajas, N. et al. Plant-mediated synthesis and interaction of ZnO against breast and prostate cancer: Review. *Results Chem.*** 9** (2024).

[62] Awashra, M. & Młynarz, P. The toxicity of nanoparticles and their interaction with cells: An in vitro metabolomic perspective. *Nanoscale Adv.* **5**, 2674–2723 (2023). 37205285 10.1039/d2na00534d PMC10186990

[63] Ali, K. et al. Bio-functionalized CuO nanoparticles induced apoptotic activities in human breast carcinoma cells and toxicity against *Aspergillus flavus*: An in vitro approach. *Process. Biochem.* **91**, 387–397 (2020).

[64] Chaudhry, G. S., Akim, M., Sung, A. & Sifzizul, T. M. T. Y. Y. Cancer and apoptosis: The apoptotic activity of plant and marine natural products and their potential as targeted cancer therapeutics. *Front. Pharmacol.* **13** (2022). 10.3389/fphar.2022.842376 PMC9399632 36034846

[65] Shamsi, H., Yari, R. & Salehzadeh, A. Biosynthesized BiFe2O4@Ag nanoparticles mediated *Scenedesmus obliquus* induce apoptosis in AGS gastric cancer cell line. *Sci. Rep.* **14**, 10284 (2024). 38704421 10.1038/s41598-024-57157-0 PMC11069558

[66] Berehu, H. M. & Patnaik, S. Biogenic zinc oxide nanoparticles synthesized from *Tinospora cordifolia* induce oxidative stress, mitochondrial damage and apoptosis in colorectal cancer. *Nanotheranostics* **8**, 312–329 (2024). 38577319 10.7150/ntno.84995 PMC10988208

[67] Nadiger, K. K. et al. Anticancer activity of *Celtis tournefortii* Lam. against human liver cancer cells. *J. Appl. Pharm. Sci.*10.7324/JAPS.2024.151332 (2024).

[68] Kumar, N. et al. Mitochondrial-mediated apoptosis as a therapeutic target for FNC (2′-deoxy-2′-b-fluoro-4′-azidocytidine)-induced Inhibition of Dalton’s lymphoma growth and proliferation. *Discov. Oncol.* **15**, 16 (2024). 38252337 10.1007/s12672-023-00829-6 PMC10803707

[69] Qiao, S. et al. Nanomaterials-induced programmed cell death: Focus on mitochondria. *Toxicology* **504**, 153803 (2024). 38616010 10.1016/j.tox.2024.153803

[70] Kumar, N. et al. Pharmacological insights: Mitochondrial ROS generation by FNC (Azvudine) in Dalton’s lymphoma cells revealed by super resolution imaging. *Cell. Biochem. Biophys.* **82**, 873–883 (2024). 38483755 10.1007/s12013-024-01238-4

[71] Chavez-Dominguez, R., Perez-Medina, M., Lopez-Gonzalez, J. S., Galicia-Velasco, M. & Aguilar-Cazares, D. The double-edge sword of autophagy in cancer: From tumor suppression to pro-tumor activity. *Front. Oncol.* **10** (2020). 10.3389/fonc.2020.578418 PMC7575731 33117715

[72] Yamaguchi, H. et al. Transforming somatic mutations of mammalian target of Rapamycin kinase in human cancer. *Cancer Sci.* **106**, 1687–1692 (2015). 26432419 10.1111/cas.12828 PMC4714661

[73] Roy, R. et al. Zinc oxide nanoparticles induce apoptosis by enhancement of autophagy via PI3K/Akt/mTOR inhibition. *Toxicol. Lett.* **227**, 29–40 (2014). 24614525 10.1016/j.toxlet.2014.02.024

[74] Alinovi, R. et al. Titanium dioxide aggregating nanoparticles induce autophagy and under-expression of MicroRNA 21 and 30a in A549 cell line: A comparative study with cobalt(II, III) oxide nanoparticles. *Toxicol. Vitro*. **42**, 76–85 (2017). 10.1016/j.tiv.2017.04.007 28400205

[75] Xu, H. et al. Zn-doped CuO nanocomposites inhibit tumor growth by NF-κB pathway cross-linked autophagy and apoptosis. *Nanomedicine* **14**, 131–149 (2019). 30394176 10.2217/nnm-2018-0366

[76] Zhang, Z., Liu, T., Yu, M., Li, K. & Li, W. The plant alkaloid tetrandrine inhibits metastasis via autophagy-dependent Wnt/β-catenin and metastatic tumor antigen 1 signaling in human liver cancer cells. *J. Exp. Clin. Cancer Res.* **37**, 1–11 (2018). 29334999 10.1186/s13046-018-0678-6 PMC5769468

[77] Liu, Y. et al. Nanotherapeutics targeting autophagy regulation for improved cancer therapy. *Acta Pharm. Sin B* **14**, 2447–2474 (2024). 38828133 10.1016/j.apsb.2024.03.019 PMC11143539

[78] Kumar, N. et al. FNC (4′-azido-2′-deoxy-2′-fluoro(arbino)cytidine) as an effective therapeutic agent for NHL: ROS generation, cell cycle arrest, and mitochondrial-mediated apoptosis. *Cell. Biochem. Biophys.* **82**, 623–639 (2024). 38253918 10.1007/s12013-023-01193-6

[79] Tomecka, P. et al. Factors determining epithelial–mesenchymal transition in cancer progression. *Int. J. Mol. Sci.* **25**, 8972 (2024). 39201656 10.3390/ijms25168972 PMC11354349

[80] Fayzullina, D. et al. Novel targeted therapeutic strategies for ewing sarcoma. *Cancers (Basel)* **14**, 1988 (2022). 35454895 10.3390/cancers14081988 PMC9032664

[81] Zhang, Z. J., Yang, Y. K. & Wu, W. Z. Bufalin attenuates the stage and metastatic potential of hepatocellular carcinoma in nude mice. *J. Transl. Med.* **12**, 57 (2014). 24581171 10.1186/1479-5876-12-57 PMC4015709

[82] Mohammadinejad, R. et al. Necrotic, apoptotic and autophagic cell fates triggered by nanoparticles. *Autophagy* **15**, 4–33 (2019). 30160607 10.1080/15548627.2018.1509171 PMC6287681

[83] Kouroumalis, E., Tsomidis, I. & Voumvouraki, A. Pathogenesis of hepatocellular carcinoma: The interplay of apoptosis and autophagy. *Biomedicines* **11**, 1–45 (2023). 10.3390/biomedicines11041166 PMC10135776 37189787

[84] Rahman, M. A. et al. Advancements in utilizing natural compounds for modulating autophagy in liver cancer: Molecular mechanisms and therapeutic targets. *Cells* **13** (2024). 10.3390/cells13141186 PMC11274515 39056768

[85] Li, X. et al. Zinc-doped copper oxide nanocomposites inhibit the growth of pancreatic cancer by inducing autophagy through AMPK/mTOR pathway. *Front. Pharmacol.* **10**, 1–11 (2019). 31001120 10.3389/fphar.2019.00319 PMC6454023

[86] Bai, D. P., Zhang, X. F., Zhang, G. L., Huang, Y. F. & Gurunathan, S. Zinc oxide nanoparticles induce apoptosis and autophagy in human ovarian cancer cells. *Int. J. Nanomed.* **12**, 6521–6535 (2017). 10.2147/IJN.S140071 PMC5592910 28919752

[87] Hashemi, M. et al. Targeting and regulation of autophagy in hepatocellular carcinoma: Revisiting the molecular interactions and mechanisms for new therapy approaches. *Cell. Commun. Signal.* **21**, 32 (2023). 36759819 10.1186/s12964-023-01053-z PMC9912665

[88] Giannelli, G., Koudelkova, P., Dituri, F. & Mikulits, W. Role of epithelial to mesenchymal transition in hepatocellular carcinoma. *J. Hepatol.* **65**, 798–808 (2016). 27212245 10.1016/j.jhep.2016.05.007

[89] Shin, S. K. et al. Clinical significance of combined epithelial–mesenchymal transition markers expression and role of Rac1 in hepatocellular carcinoma. *Int. J. Mol. Sci.* **24**, 1–15 (2023). 10.3390/ijms24021765 PMC9865966 36675278

[90] Ashrafizadeh, M. et al. New insight towards development of paclitaxel and docetaxel resistance in cancer cells: EMT as a novel molecular mechanism and therapeutic possibilities. *Biomed. Pharmacother*. **141**, 111824 (2021). 34175815 10.1016/j.biopha.2021.111824

[91] Ashrafizadeh, M. et al. Role of microRNA/epithelial-to-mesenchymal transition axis in the metastasis of bladder cancer. *Biomolecules* **10**, 1159 (2020). 32784711 10.3390/biom10081159 PMC7464913

[92] MIMA, K. et al. Epithelial–mesenchymal transition expression profiles as a prognostic factor for disease-free survival in hepatocellular carcinoma: Clinical significance of transforming growth factor-β signaling. *Oncol. Lett.* **5**, 149–154 (2013). 23255911 10.3892/ol.2012.954 PMC3525349

[93] Chen, S., Liao, Z. & Xu, P. Mitochondrial control of innate immune responses. *Front. Immunol.*** 14** (2023). 10.3389/fimmu.2023.1166214 PMC10267745 37325622

