# Supplementary material for: Unveiling the potency of ZnO and CuO nanocomposites in combating hepatocellular carcinoma by inducing cell death and suppressing migration
Source: Sci Rep. 2025 May 3;15:15477. doi: 10.1038/s41598-025-97395-4 (PMC12049527; doi:10.1038/s41598-025-97395-4)
Supplement: Supplementary file 1 — Supplementary Material 1 [file 41598_2025_97395_MOESM1_ESM.pdf]

Beclin-1

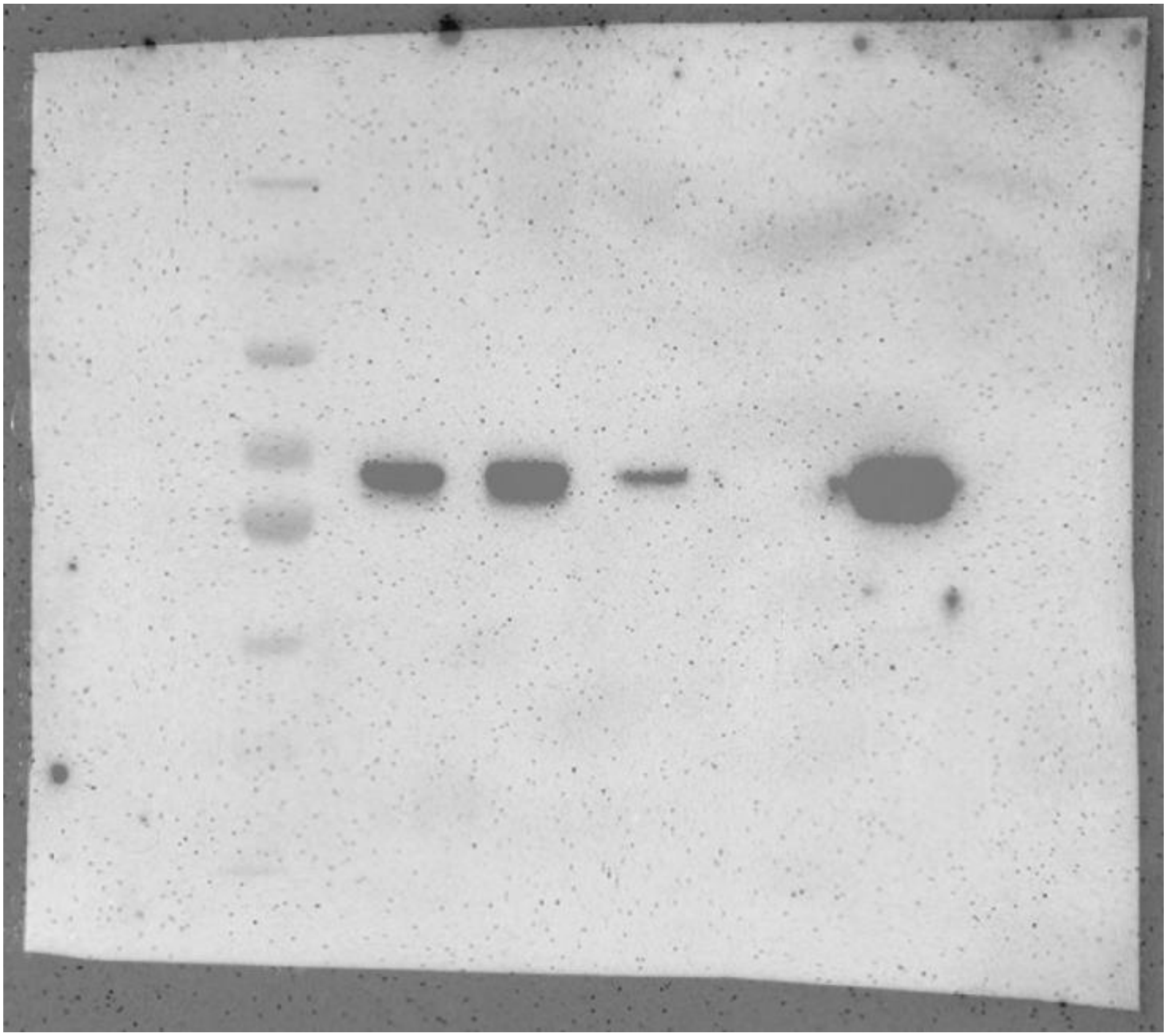

Figure I: Autophagic cell death evaluation in HuH-7 cells after exposure to N1 and sorafenib for 48 h.

Vimentin

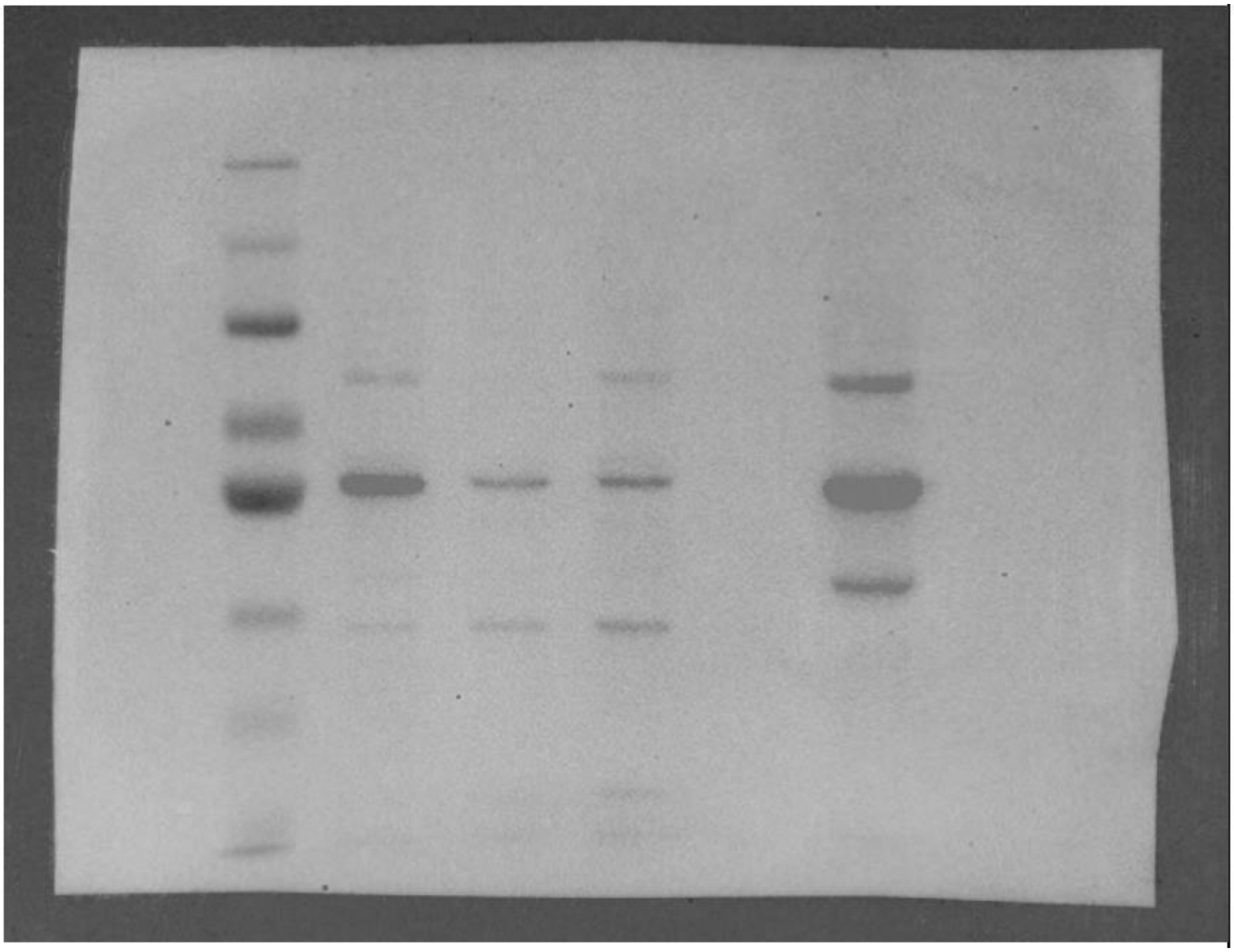

Figure II: Antimigratory activity of N1 and Sorafenib on the migration of HuH-7.

GAPDH

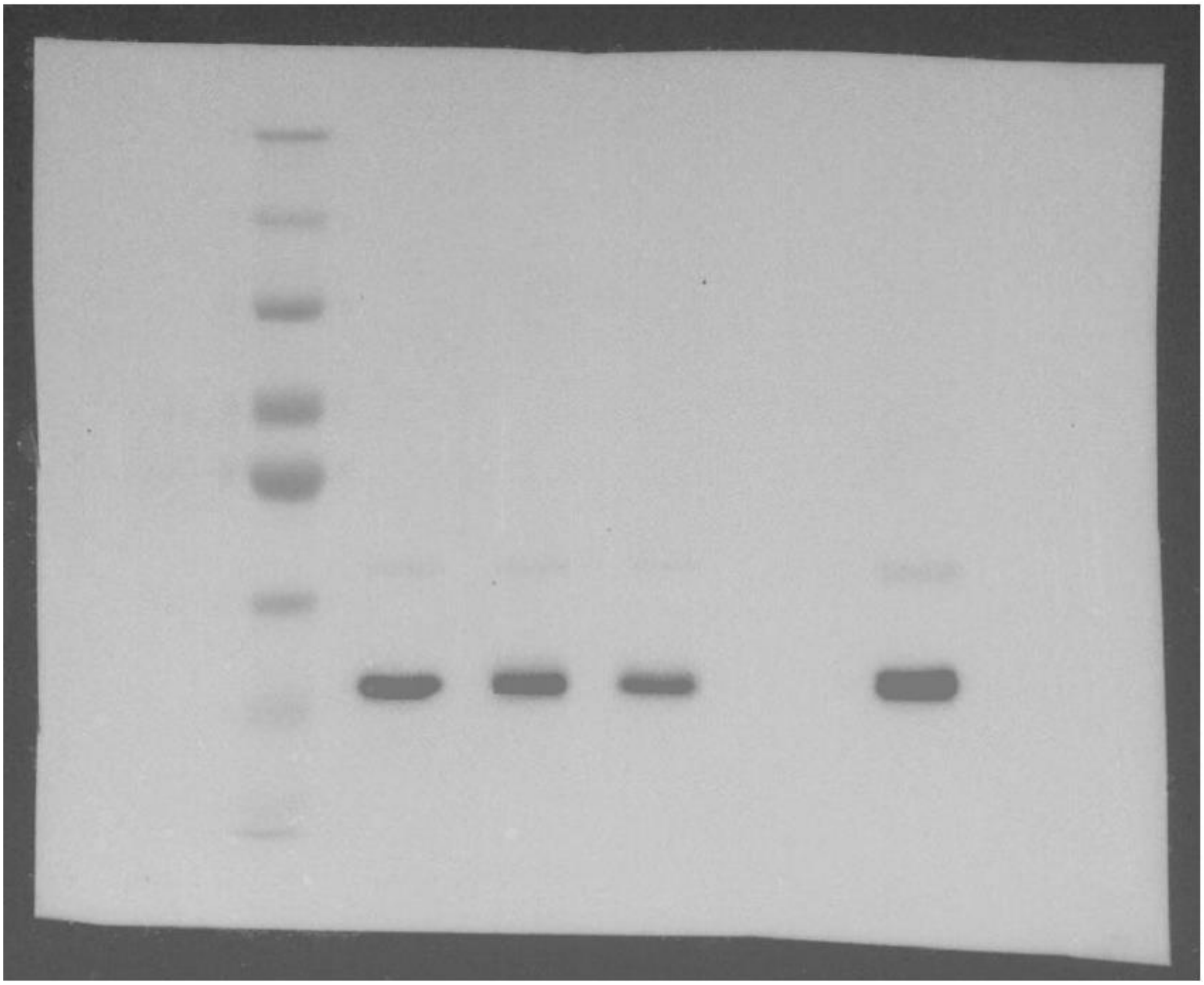

Figure III: Autophagic cell death evaluation in HuH-7 cells after exposure to N1 and sorafenib for 48 h.
